# Interventions on cognitions and perceptions that influence work participation of employees with chronic health problems: a scoping review

**DOI:** 10.1186/s12889-020-09621-5

**Published:** 2020-10-27

**Authors:** Mariska De Wit, Bedra Horreh, Joost G. Daams, Carel T. J. Hulshof, Haije Wind, Angela G. E. M. de Boer

**Affiliations:** Department of Public and Occupational Health, Coronel Institute of Occupational Health, Amsterdam Public Health research institute, Amsterdam UMC, University of Amsterdam, PO Box 22700, 1100 DE Amsterdam, The Netherlands

**Keywords:** Occupational health professionals, Person-related factors, Cognitions, Perceptions, Return to work

## Abstract

**Background:**

Cognitions and perceptions, such as motivation and return to work (RTW) expectations, can influence work participation of employees with chronic health problems. This makes these cognitions and perceptions important factors for occupational health professionals to intervene upon in order to increase work participation. There is, however, no overview of interventions that influence these factors and are aimed at increasing work participation. Therefore, the purpose of this scoping review is to explore available interventions that are focused on cognitions and perceptions of employees with chronic health problems and aimed at increasing work participation.

**Methods:**

A scoping review was carried out following the framework of Arksey and O’Malley. Ovid MEDLINE and PsycINFO were searched for original papers published between January 2013 and June 2020. We included studies that describe interventions that focus on at least one of ten cognitions and perceptions and on work participation. The risk of bias of the studies included was assessed using quality assessment tools from the Joanna Briggs Institute.

**Results:**

In total, 29 studies were identified that studied interventions aimed at changing at least one of ten cognitions and perceptions in order to change work participation. The interventions that were included mainly focused on changing recovery and RTW expectations, self-efficacy, feelings of control, perceived health, fear-avoidance beliefs, perceived work-relatedness of the health problem, coping strategies and catastrophizing. No interventions were found that focused on changing motivation or on optimism/pessimism. Four interventions were judged as effective in changing coping, self-efficacy, fear-avoidance beliefs, or perceived work-relatedness and work participation according to results of randomized controlled trials.

**Conclusions:**

This review provides an overview of interventions that focus on changing cognitions and perceptions and work participation. Evidence was found for four effective interventions focused on changing these factors and increasing work participation. Occupational health professionals may use the overview of interventions to help employees with chronic health problems to increase their work participation.

## Background

Occupational health professionals (OHPs) play an important role in increasing work participation in employees with chronic health problems. By OHPs, we refer to all professionals who make decisions about work participation or about receiving benefits for employees with health problems. In their practice it is important for them to focus on factors that may influence the work participation of these employees.

According to the International Classification of Functioning, Disability and Health (ICF model) different domains of factors can influence a person’s work ability: disease-related factors, external factors and personal factors [1]. Personal factors that can influence work participation are cognitions and perceptions of employees [2–4]. In contrast to some other factors, cognitions and perceptions of employees are not always easy to recognize by OHPs. In addition, some employees may not even be aware that they have cognitions and perceptions that limit their work participation. In a study by De Wit et al. [2], six cognitions and perceptions were identified that were positively associated with work participation: positive recovery and return to work (RTW) expectations, optimism, self-efficacy, motivation, feelings of control, and perceived health. Four cognitions and perceptions were negatively associated with work participation: fear-avoidance beliefs, perceived work-relatedness of the health problem, limiting coping strategies and catastrophizing [2]. The association between these ten cognitions and perceptions and work participation makes them important targets for intervention.

To promote work participation in employees with chronic health problems, relevant cognitions and perceptions should be identified. Next, the hindering cognitions and perceptions should be limited and the positive cognitions and perceptions fostered [2].

To help employees who have cognitions and perceptions that can negatively influence work participation or to foster positive cognitions and perceptions, it is important for OHPs to get an overview of available interventions that may help to influence these factors. OHPs can recommend these interventions in order to increase work participation. However, as far as we know, no such a review about these interventions exists. Therefore, the purpose of this scoping review is to explore available interventions that are focused on at least one of the cognitions and perceptions and aimed at increasing work participation of employees with chronic health problems. The main question for this study is: Which interventions are available that are focused on cognitions and perceptions and aimed at increasing work participation of employees with chronic health problems?

## Methods

### Methodology

To answer our research question, we conducted a scoping review. We chose for a scoping review, because in contrast to a systematic review we do not have a focused research question on finding evidence for an association between variables. Instead, we have a broad and explorative research question about available interventions. In addition, we aim to summarize and disseminate our research findings to physicians and to consult physicians and patient representatives to get feedback on our findings, which is an essential component of scoping reviews [5].

We used the Joanna Briggs Institute Reviewers’ Manual for methodology for Scoping Reviews [6] and the scoping review framework of Arksey and O’Malley [7] for conducting the review. This framework consists of six stages for conducting a scoping review: 1) identifying the research question, 2) identifying relevant studies, 3) study selection, 4) charting the data, 5) collating, summarizing and reporting the results, and 6) consultation. We used the PRISMA Extension for Scoping Reviews (PRISMA-ScR) Checklist for making sure that we reported all the relevant components of this scoping review [8].

### Identifying the research question

The main question of this scoping review, as identified in the introduction is: Which interventions are available that are focused on cognitions and perceptions and aimed at increasing work participation of employees with chronic health problems?

### Identifying relevant studies

The search strategy was developed with the help of a research librarian (JD). In order to find relevant words in titles and abstracts that can be used in the full search strategy, we first performed a limited search in Ovid MEDLINE to identify relevant articles. The complete search strategy consists of terms related to three elements of the PICO. In this review the population (P) are employees of working age (18–67 years) with chronic health problems. We defined chronic health problems according to the definition of the World Health Organization: Diseases with long duration and generally slow progression [9]. The interventions (I) in this review are interventions that focus on at least one of the ten cognitions and perceptions that are associated with work participation: expectations regarding recovery or RTW, optimism/pessimism, self-efficacy, motivation, feelings of control, perceived health, fear-avoidance beliefs, perceived work-relatedness, catastrophizing and coping strategies [2]. In this review, work participation is the outcome (O), and this covers concepts such as RTW, sickness absence and current work status. With the full search strategy we looked for relevant articles in Ovid MEDLINE and PsycINFO. The two search strategies are presented in Additional file 1.

#### Inclusion criteria

Only studies recently published, between January 1st 2013 and June 15th 2020, in peer-reviewed journals were included. Cohort studies, (randomized) controlled trials, and studies with pre-test post-test designs were included. Reference lists from relevant reviews and meta-analyses we found were screened for additional relevant studies. Articles were only considered eligible for inclusion if they were available in English or Dutch.

#### Exclusion criteria

Case studies and qualitative studies were excluded from this review. We also excluded articles in which participants are younger than 18 or older than 67 years, are students, are military personnel or veterans, are volunteers (no paid job) or are employees with substance abuse problems.

### Study selection

For identifying and selecting relevant studies, we used the web application Rayyan [10]. The title and abstract of all records were independently screened on relevance based on previously identified inclusion and exclusion criteria by two reviewers (MdW and HW, MdW and CH, MdW and AdB or MdW and BH). For every excluded article, at least one reason for exclusion was reported by the researchers. If there was disagreement about possible relevance of these studies, the reasons for exclusion were discussed by the researchers until consensus was reached about inclusion or exclusion. If the researchers thought the article was potentially relevant, the full article was read and independently screened for relevance by two reviewers (MdW and BH). Disagreements about inclusion of the studies after reading the full text were discussed with all researchers until consensus was reached about inclusion or exclusion. The reference lists of reviews and meta-analyses that were found were independently screened for additional relevant studies by two reviewers and possible relevance of these studies was discussed (MdW and BH).

### Charting the data

For data charting we used a charting table drawn up by the research team. In this table, the following characteristics of the studies included in the review were described: first author, year of publication, country, study design, characteristics of study population (number of participants, mean age, gender, health status) and intervention types (duration, number and type of sessions, providers of the intervention, main components of the intervention). In addition, we described the cognitions and perceptions in that study, how they are measured and the follow-up period. Finally, we described the effect of the intervention on the cognition or perception of interest and on work participation. The data were charted by two researchers (MdW and BH). All data charting was discussed between the two researchers until consensus was reached. After this, the other researchers (AdB, HW, CH) each checked one third of the data-extraction, so that all data were ultimately checked.

### Collating, summarizing and reporting the results

We assessed the quality of the studies with the assessment instruments of the Joanna Briggs Institute, which has different criteria for different study types, and we presented the scores in tables [11]. The detailed characteristics of the studies are presented in the Additional file. We presented the effects of the interventions from the eligible studies per factor in two tables, one table for interventions that were studied in randomized controlled trials (RCTs) and one table for interventions that were studied with other study designs. In these tables we presented the health problems of the study population, the name and type of the intervention of interest, and the effect of the intervention on the cognition or perception and on work participation. We also reported whether, based on the findings in our review, the intervention should be recommended by OHPs.

### Consultation

The last stage in the framework of Arksey and O’Malley [7] is the consultation of stakeholders. We consulted OHPs and a patient representative by e-mail or in a face-to-face meeting to obtain feedback on the findings. In the Netherlands the two important groups of OHPs are occupational physicians (OPs) and insurance physicians (IPs). OPs focus particularly on prevention of work-related diseases, health promotion, and in guiding employees with health problems in their RTW or in retaining work. IPs try to help to increase work participation in these employees by evaluating the functional abilities of the employee and by determining whether employees should receive a work disability benefit. We asked the OPs, IPs and patient representative about their experience with the interventions or components of the interventions and what to consider when a physician wants to recommend the interventions in daily practice. During the face-to-face meeting notes were made by the researcher (MdW). The most important notes and the answers by email were summarized by one researcher (MdW) and checked by the other researchers (AdB, CH, and HW). We used the feedback from the OPs, IPs and patient representative to describe the implications for practice in order to make the results of this study more practical for OHPs.

## Results

### Studies selected

**Fig. 1:**
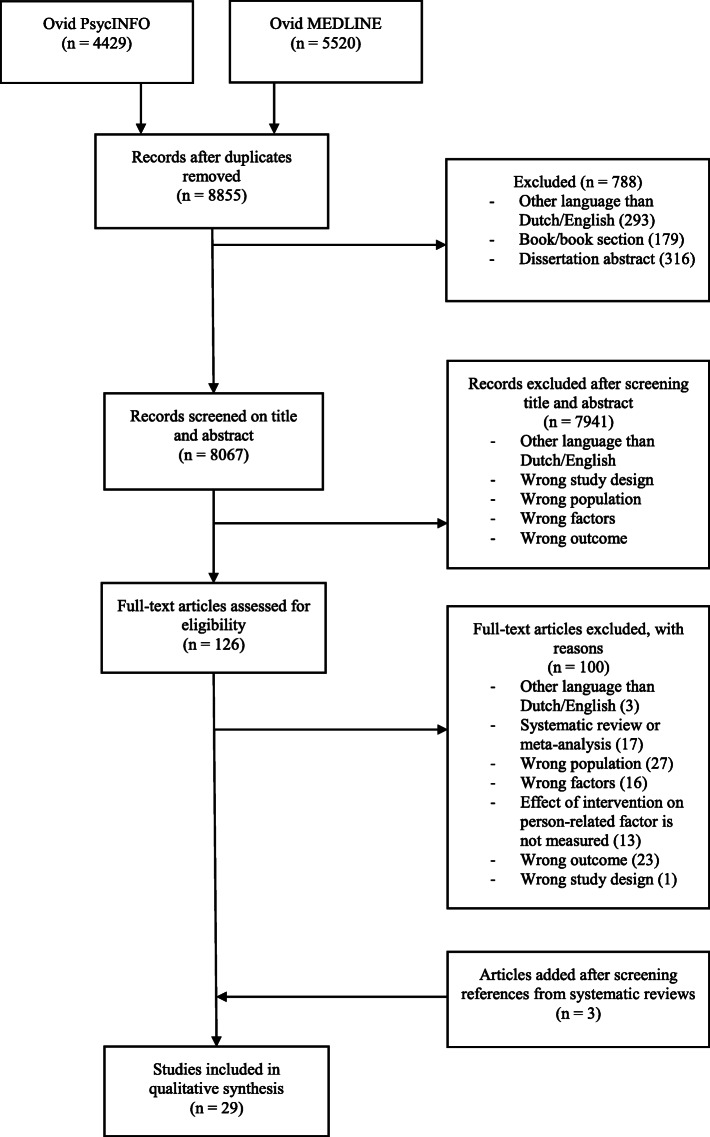
Flow-chart of the search process

The search process is presented in Fig. 1. In total, 4429 studies were found in PsycINFO and 5520 studies in Ovid MEDLINE. Twenty-nine studies were included in this review. The final sample consisted of sixteen RCTs, nine cohort studies, three studies with a single group pre-test post-test design and one non-randomized experimental study.

Table 1 and Table 2 describe the effect of the interventions in question on cognitions and perceptions and on work participation. They also indicate whether OHPs should recommend the intervention to employees – a matter that remains unclear for a couple of interventions because the effects of the interventions are not compared between an intervention and a control group. Detailed characteristics of the final studies that were included in this review are presented in Additional file 2.

**Table 1:** Effect of interventions studied in randomized controlled trials

Study	Health problem population	Name of intervention	Intervention type	Significant positive effect on cognition or perception compared to control group	Significant effect on work participation compared to control group	Should OHPs recommend this intervention?
**Self-efficacy**						
Hampel P. et al., 2019 [12]	Chronic low back pain	Combined cognitive behavioral pain competence and depression prevention training	Intervention including standard multidisciplinary rehabilitation, pain competence training, depression prevention training and homework assignments	**Yes**^**a**^	**Yes**	**Yes**
Hees H. L. et al., 2013 [13]	Major depressive disorder	Occupational therapy	Intervention focused on problem clarification, coping with stressors and making a re-integration plan	No	No	No
Hutting N. et al., 2015 [14]	Chronic non-specific complaints of the arm, neck or shoulder	Self-management intervention	Intervention focused on setting targets for behavior and making action plans including an eHealth module about self-management	No	No	No
Muschalla B. et al., 2016 [15]	Orthopedic disorders, cardiologic disorders, neurological disorders	Cognitive behavioral group intervention on work-anxiety	Intervention focused on problem solving, situation and behavior analysis and developing and training coping strategies	No	Yes^a^	No
Wormgoor M. E. A. et al., 2020 [16]	Common mental complaints	Brief psychotherapy	Intervention focused on normalizing, accepting and coping with mental health complaints and their hindrance for work participation	No	Yes^b^	No
**Perceived health**						
Fauser D. et al., 2019 [17]	Cancer	Conventional medical rehabilitation plus additional work-related modules	Intervention including exercise therapy, occupational therapy, psychological counseling and work-related functional capacity training	No	No	No
Pedersen P. et al., 2015 [18]	Anxiety, depression, other mental illness, stress and burnout, musculoskeletal disorders	Psychoeducation	Intervention with lectures and discussions about problem solving techniques and coping strategies and sessions with relatives	No	No	No
Van Eijk-Hustings Y. et al., 2013 [19]	Fibromyalgia	Multidisciplinary intervention with aftercare	Intervention consisting of sociotherapy, physiotherapy, psychotherapy, creative therapy and an aftercare program	No	No	No
**Feelings of control**						
Muschalla B. et al., 2016 [15]	Orthopedic disorders, cardiologic disorders, neurological disorders	Cognitive behavioral group intervention on work-anxiety	Intervention focused on problem solving, situation and behavior analysis and developing and training coping strategies	No	Yes^a^	No
Pedersen P. et al., 2015 [18]	Anxiety, depression, other mental illness, stress and burnout, musculoskeletal disorders	Psychoeducation	Intervention with lectures and discussions about problem solving techniques and coping strategies and sessions with relatives	Yes^c^	No	No
**Catastrophizing**						
Hutting N. et al., 2015 [14]	Chronic non-specific complaints of the arm, neck or shoulder	Self-management intervention	Intervention focused on setting targets for behavior and making action plans including an eHealth module about self-management	No	No	No
Rolving N. et al., 2015 [20]	Degenerative disc disease or spondylolisthesis	Cognitive behavioral therapy	Intervention with group discussions about pain perception, coping, pacing principles and RTW including homework assignments about thoughts and feelings in relation to stressful situations, coping strategies, and setting goals	Yes^d^	No	No
**Fear-avoidance beliefs**						
Aasdahl L. et al., 2019 [21]	Musculoskeletal, psychological or general and unspecified diagnosis	Short inpatient program	Intervention including acceptance and commitment therapy, physical training, mindfulness, psychoeducation, meetings with an employer and making a RTW plan	No	–	No
Aasdahl L. et al., 2019 [21]	Musculoskeletal, psychological or general and unspecified diagnosis	Long inpatient program	Intervention including acceptance and commitment therapy, physical training, mindfulness, psychoeducation, outdoor activities, a network day and making a RTW plan	No	–	No
Granviken F. et al., 2015 [22]	Subacromial impingement	Supervised exercise intervention	Intervention with a theory lesson about rehabilitation, supervised exercise therapy focused on movement patterns and home exercises	No	No	No
Harris A. et al., 2017 [23]	Non-specific low back pain	Group physical exercise	Intervention with physical exercise in groups and sessions about coping, chronic pain and ergonomics	No	No	No
Harris A. et al., 2017 [23]	Non-specific low back pain	Group cognitive behavioral therapy	Intervention with homework consisting of exposure to pain-provoking physical activity and group discussions to change dysfunctional thoughts	No	No	No
Marchand G. H. et al., 2015 [24]	Neck and/or back pain	Work-focused intervention	Intervention including contact with a caseworker about work and obstacles to RTW and creating a RTW schedule	No	No	No
Ronzi Y. et al., 2017 [25]	Non-specific chronic low back pain	Ambulatory individual physiotherapy	Intervention with individual sessions with active exercises supervised by a physiotherapist and home exercises	No	No	No
Ronzi Y. et al., 2017 [25]	Non-specific chronic low back pain	Mixed strategy	Intervention with individual and group sessions with physiotherapy and discussions about beliefs and meetings with a psychologist	No	No	No
Vibe Fersum K. et al., 2013 [26]	Non-specific chronic low back pain	Classification-based cognitive functional therapy	Intervention with focus on outlining the vicious cycle of pain based on examination findings, movement exercises and tailored physical activity	**Yes**	**Yes**	**Yes**
**Perceived work-relatedness**						
Muschalla B. et al., 2016 [15]	Orthopedic disorders, cardiologic disorders, neurological disorders	Cognitive behavioral group intervention on work-anxiety	Intervention focused on problem solving, situation and behavior analysis and developing and training coping strategies	**Yes**	**Yes**^**a**^	**Yes**
**Coping strategies**						
Arends I. et al., 2014 [27]	Common mental disorders	Stimulating healthy participation and relapse prevention at work intervention	Intervention focused on the process of problem solving including inventory of problems, brainstorming about solutions and making an action plan	**Yes**^**c,d**^	**Yes**	**Yes**
Fauser D. et al., 2019 [17]	Cancer	Conventional medical rehabilitation plus additional work-related modules	Intervention including exercise therapy, occupational therapy, psychological counseling and work-related functional capacity training	No	No	No
Harris A. et al., 2017 [23]	Non-specific low back pain	Group physical exercise	Intervention with physical exercise in groups and sessions about coping, chronic pain and ergonomics	No	No	No
Harris A. et al., 2017 [23]	Non-specific low back pain	Group cognitive behavioral therapy	Intervention with homework consisting of exposure to pain-provoking physical activity and group discussions to change dysfunctional thoughts	No	No	No
Hees H. L. et al., 2013 [13]	Major depressive disorder	Occupational therapy	Intervention focused on problem clarification, coping with stressors and making a re-integration plan	No	No	No
Muschalla B. et al., 2016 [15]	Orthopedic disorders, cardiologic disorders, neurological disorders	Cognitive behavioral group intervention on work-anxiety	Intervention focused on problem solving, situation and behavior analysis and developing and training coping strategies	**Yes**^**c**^	**Yes**^**a**^	**Yes**

*RTW Return to work, *OHPs* Occupational health professionals

Underlined studies had a low risk of bias, bold studies describe interventions that should be recommended by OHPs

^a^Depends on population characteristics

^b^Not for every moment on which the outcome is measured

^c^Depends on the form/subscale of the factor

^d^Not for every moment on which the cognition or perception is measured

**Table 2:** Effect of interventions studied in cohort studies, non-randomized experimental studies and pre-test post-test studies

Study	Health problem population	Name of Intervention	Intervention type	Significant positive effect on cognition or perception over time	Significant positive effect on work participation over time	Significant positive effect on work participation through change in cognition or perception	Should OHPs recommend this intervention?
**Self-efficacy**							
Chu M. C. et al., 2015 [28]	Chronic non-cancer pain	Comprehensive outpatient pain engagement program	Intervention with education about pain pathophysiology, behavioral training, exercises, thought management and activity planning	Yes	Yes	–	Unclear
Jensen A. G. C., 2013 [29]	Mental illness, musculoskeletal illness, mental and musculoskeletal illness	RTW intervention	Intervention including making an individually tailored rehabilitation plan, physical exercises, an ergonomic course and cognitive therapy	No	Yes^a^	Yes	No
Leensen M. C. J. et al., 2017 [30]	Cancer	Multidisciplinary rehabilitation program	Intervention with supervised interval and resistance exercises and counseling sessions with advice on work resumption	Yes^b^	Yes	–	Unclear
Salzwedel A. et al., 2020 [31]	Cardiovascular diseases	Standardized comprehensive cardiac rehabilitation	Intervention including risk-factor modification, exercise training, psychosocial interventions, vocational assessment and counseling	Yes	NR	–	Unclear
**Perceived health**							
Chu M. C. et al., 2015 [28]	Chronic non-cancer pain	Comprehensive outpatient pain engagement program	Intervention with education about pain pathophysiology, behavioral training, exercises, thought management and activity planning	Yes	Yes	–	Unclear
Haiduk P. et al., 2017 [32]	Chronic neck pain	The 4 interdisciplinary pain program	Intervention with physiotherapy, strength training, occupational therapy, cognitive behavioral and coping therapy and relaxation	Yes^b^	NR	–	Unclear
Jensen A. G. C., 2013 [29]	Mental illness, musculoskeletal illness, mental and musculoskeletal illness	RTW intervention	Intervention including making an individually tailored rehabilitation plan, physical exercises, an ergonomic course and cognitive therapy	No	Yes^a^	Yes	No
Pietilä-Holmner E. et al., 2020 [33]	Chronic musculoskeletal pain	Multimodal rehabilitation program	Intervention with physical exercise, relaxation, education in pain management and training in coping strategies based on cognitive behavioral therapy	Yes	Yes	–	Unclear
**Recovery or RTW expectations**							
Aasdahl L. et al., 2018 [34]	Musculoskeletal, psychological or general and unspecified diagnoses	Short inpatient program, long inpatient program, outpatient program	Interventions including acceptance and commitment therapy, physical training, mindfulness, psycho-education, problem solving sessions and making a RTW plan	Yes	NR	Yes	Unclear
**Catastrophizing**							
Adams H. et al., 2017 [35]	Major depressive disorder	Risk-targeted activity-reintegration intervention/Progressive goal attainment program	Intervention focused on goal setting, activity planning and learning techniques for targeting disability beliefs including problem solving challenges and exposing techniques	Yes	NR	Yes	Unclear
Chu M. C. et al., 2015 [28]	Chronic non-cancer pain	Comprehensive outpatient pain engagement program	Intervention with education about pain pathophysiology, behavioral training, exercises, thought management and activity planning	Yes	Yes	–	Unclear
Gagnon C. M. et al., 2013 [36]	Chronic pain	Interdisciplinary pain management program	Intervention with psychological treatment, occupational therapy, physical therapy and vocational counseling	Yes	Yes	–	Unclear
Haiduk P. et al., 2017 [32]	Chronic neck pain	The 4 interdisciplinary pain program	Intervention with physiotherapy, strength training, occupational therapy, cognitive behavioral and coping therapy and relaxation	Yes	NR	–	Unclear
Pietilä-Holmner E. et al., 2020 [33]	Chronic musculoskeletal pain	Multimodal rehabilitation program	Intervention with physical exercise, relaxation, education in pain management and training in coping strategies based on cognitive behavioral therapy	Yes	Yes	–	Unclear
Scott W. et al., 2014 [37]	Whiplash injury	Multidisciplinary rehabilitation program	Intervention with tailored exercises, education and instruction in self-management skills	NR	NR	Yes	Unclear
Sullivan M. et al., 2017 [38]	Post-traumatic stress disorder	Risk-targeted activity-reintegration intervention/Progressive goal attainment program	Intervention focused on goal setting, activity planning and learning techniques for targeting disability beliefs including problem solving challenges and exposing techniques	Yes	NR	Yes	Unclear
Volker G. et al., 2017 [39]	Chronic musculoskeletal pain	Standardized multidisciplinary team care intervention	Intervention with cognitive behavioral therapy, exercises, relaxation and education	Yes	Yes	–	Unclear
**Coping**							
Asih S. et al., 2015 [40]	Chronic disabling occupational musculoskeletal disorders	Functional restoration program	Intervention with cognitive behavior therapy, coping skills training, fear-avoidance beliefs training and exercises	Yes	NR	Yes^c^	Unclear
Pietilä-Holmner E. et al., 2020 [33]	Chronic musculoskeletal pain	Multimodal rehabilitation program	Intervention with physical exercise, relaxation, education in pain management and training in coping strategies based on cognitive behavioral therapy	Yes	Yes	–	Unclear

* *RTW* Return to work, *OHPs* Occupational health professionals, *NR* Statistical significance not reported

Underlined studies had a low risk of bias

^a^Not for every moment on which the outcome is measured

^b^Not for every moment on which the cognition or perception is measured

^c^Depends on the form/subscale of the outcome

### Risk of bias

Fifteen of the sixteen RCTs had a moderate risk of bias and one had a low risk of bias. Five of the cohort studies had a moderate risk of bias, one had a high risk of bias and three had a low risk of bias. Of the non-randomized experimental studies and single group pre-test post-test studies, there were three with a moderate risk of bias and one with a low risk of bias. Scores on each criterion of the quality assessment tools are presented in Additional file 3.

### Factors positively associated with work participation

#### Self-efficacy

Nine studies, of which five were RCTs, studied the effect of an intervention on self-efficacy and work participation [12–16, 28–31]. The RCT of Hees et al. [13], which was described in detail in Hees et al. [41], the RCT of Hutting et al. [14], the RCT of Muschalla et al. [15] and the RCT of Wormgoor et al. [16] did not show a significant effect on self-efficacy. Only the “Combined cognitive behavioral pain competence and depression prevention training” described in the RCT of Hampel et al. [12] increased self-efficacy in participants with chronic low back pain and high levels of depressive symptoms. This intervention also resulted in a decrease in days of sick leave and had a positive effect on employment status. The intervention consisted of eight group sessions focused on for example treating pain-related beliefs, pain management, enhancement of activities and social skills training. The cohort study by Chu et al. [28] among employees with non-cancer pain and the study of Leensen et al. [30] among employees with cancer both showed a positive effect on self-efficacy and on work participation. These interventions were multidisciplinary interventions, which included exercises from physiotherapists and sessions directed to activity planning or planning for gradually resuming work. The difference between these interventions was that one of them consisted mostly of individual sessions over a longer period of twelve weeks [30], while the other consisted of group sessions over a shorter period of fourteen days [28]. Although both studies showed a positive effect of the intervention on self-efficacy and on work participation, the researchers of these studies did not study whether change in work participation was caused by the change in self-efficacy. In addition, the intervention described by Salzwedel et al. [31] among employees with a cardiovascular disease had a positive effect on self-efficacy. However, the statistical significance of the effect on work status was not reported. The intervention in the study of Jensen [29] among employees with mental or musculoskeletal illness, which was more precisely described by Jensen [42], showed no effect on self-efficacy.

#### Perceived health

Seven studies, of which three RCTs, studied the effect of an intervention on perceived health and work participation [17–19, 28, 29, 32, 33]. The interventions of Pedersen et al. [18], Fauser et al. [17] and Van Eijk-Hustings et al. [19] did not have a significant effect on perceived health. The intervention in the cohort study of Chu et al. [28] on thought management and activity planning among employees with chronic non-cancer pain increased perceived health and improved the work status of employees. However, no results were reported regarding whether the increase in perceived health caused the increase in work participation. In addition, the intervention in the cohort of Pietilä-Holmner et al. [33] with physical exercise, education in pain management and training coping strategies, increased perceived health and decreased sick leave among employees with chronic musculoskeletal pain. However, they did not report whether the increase in perceived health caused the increase in work participation either. Also, the intervention in the study of Haiduk et al. [32] among employees with chronic neck pain showed a significant positive effect on perceived health after 60 months. It seemed to increase working capacity, although the statistical significance of this last effect was not reported. This intervention focused on strength training, occupational therapy, cognitive behavioral therapy and coping therapy. The intervention in a cohort study of Jensen [29] did not have a significant effect on perceived health.

#### Recovery and RTW expectations

One study of Aasdahl et al. [34] studied the effect of an intervention on RTW expectations and work participation among employees with different kinds of chronic diseases. The intervention involved acceptance and commitment therapy, physical training and psycho-education. This intervention significantly improved the expectations of employees regarding RTW. In this study, the improvement in these expectations was associated with sustainable RTW and more work participation days.

#### Motivation

No studies were found on interventions that were focused on motivation and aimed at increasing work participation.

#### Optimism

No studies were found on interventions that were focused on optimism or pessimism and aimed at increasing work participation.

#### Feelings of control

Two RCTs with interventions focused on feelings of control and work participation were found [15, 18]. The intervention of Muschalla et al. [15] did not have an effect on internal and external control perception. However, the intervention studied by Pedersen et al. [18], which was directed to problem solving techniques and coping strategies, did show that internal locus of control was higher for employees in the intervention group at three and six months follow-up in comparison with the control group. There were no differences in other locus of control variables. However, at three months, more participants in the control group than in the intervention group had full RTW, which indicates a negative effect of the intervention on work participation. There were no significant differences in RTW between the intervention and the control group at six or twelve months.

### Factors negatively associated with work participation

#### Catastrophizing

Most of the studies we found which focused on cognitions and perceptions and work participation were aimed at the factor catastrophizing. In total, ten studies were found that focused on this factor and work participation [14, 20, 28, 32, 33, 35–39]. Among these studies there were two RCTs [14, 20]. None of the interventions that were studied in these randomized controlled trials (RCTs) had a positive effect on work participation. Only the cognitive behavioral therapy intervention of Rolving et al. [20] on pain perception, coping and pacing principles, among employees with degenerative disc disease or spondylolisthesis, which was further described in the study of Rolving et al. [43], decreased catastrophizing more in the intervention group than in de control group after six months, but not after three months and one-year follow-up. All the interventions in the other studies [28, 32, 33, 35–39] seemed to decrease catastrophizing over time, although the significance of this decrease due to the intervention on self-management skills described by Scott et al. [37], was not reported. The interventions described by Chu et al. [28] among employees with chronic non-cancer pain, Gagnon et al. [36] among employees with chronic pain, Pietilä-Holmner et al. [33] among patients with chronic musculoskeletal pain and Volker et al. [39], which was among employees with chronic musculoskeletal pain as well, significantly increased work participation over time. All these interventions had group sessions with psychological components, such as psychological treatment, thought management and cognitive behavioral therapy, and physical components, such as pool therapy and physical exercises. The interventions of Volker et al. [39], Pietilä-Holmner et al. [33] and Chu et al. [28] contained relaxation exercises as well. The multidisciplinary intervention of Haiduk et al. [32] among employees with chronic neck pain, which contained components of strength training, occupational therapy, cognitive behavioral therapy and coping therapy, seemed to increase work participation as well, although the statistical significance of this effect is not reported. In addition, the studies of Adams et al. [35], Scott et al. [37], and Sullivan et al. [38], showed that a decrease in catastrophizing was associated with a higher rate of RTW or occupational re-engagement. The “Risk-targeted activity-reintegration intervention” (or “Progressive goal attainment program”) described by Adams et al. [35] and Sullivan et al. [38], which was further described in the article of Sullivan et al. [44], consisted of maximum ten sessions focused on goal setting, activity planning, learning specific techniques to target and reduce catastrophic thinking and exposing techniques to facilitate re-engagement in activities.

#### Fear-avoidance beliefs

Six RCTs were found about interventions focused on fear-avoidance beliefs and work participation [21–26]. Only one of the studied interventions had a significant effect on this factor and on work participation [26]. The “Classification-based cognitive functional therapy” studied by Vibe Fersum et al. [26] among employees with non-specific chronic low back pain significantly decreased fear-avoidance beliefs and decreased the number of sick leave days. This intervention contained components of movement exercises, tailored physical activity and was directed at outlining the vicious cycle of pain. None of the interventions in other studies showed a significant effect on fear-avoidance beliefs as compared to the control groups [21–25].

#### Perceived work-relatedness

One RCT of Muschalla et al. [15] was found with an intervention focused on perceived work-relatedness of the health problem and work participation. This intervention, which focused mainly on developing and training coping strategies among employees with orthopedic, cardiologic and neurological disorders, decreased perceived work-relatedness in the intervention group. The intervention also reduced the sick leave duration after six months for patients with work-anxiety, but not for the whole group of participants.

#### Coping strategies

Seven studies described interventions on coping strategies and work participation, of which five RCTs and two cohort studies [13, 15, 17, 23, 27, 33, 40]. The “Stimulating health participation and relapse prevention at work” intervention of Arends et al. [27] among employees with common mental disorders and the “Cognitive behavioral group intervention on work-anxiety” of Muschalla et al. [15] among employees with orthopedic, cardiologic and neurological disorders changed coping and improved work participation. In the study of Arends et al. [27] on the effect of an intervention focused on the problem solving process, employees in the intervention group used the coping strategy distraction more often than the control group and had a lower incidence of recurrent sickness absence. However, there were no differences between the control group and intervention group in other coping strategies. In the study of Muschalla et al. [15], employees in the intervention group showed a significant increase in the coping strategies self-calming and self-instruction and showed a decrease in sick leave duration. This intervention was also directed at problem solving and contained training on strategies to cope with work-anxiety and situation and behavior analyses. The interventions studied in the RCTs by Harris et al. [23], Hees et al. [13] and Fauser et al. [17] did not significantly change coping or work participation. The intervention in the cohort study of Asih et al. [40] among employees with chronic musculoskeletal disorders significantly changed coping profiles. The intervention contained components of strength training, cognitive behavior therapy, coping skill training and fear-avoidance beliefs training. After the intervention, there were more adaptive copers and less dysfunctional copers or interpersonally distressed persons. There was a significant association between the coping profiles at discharge and work retention, but not with RTW rate. In addition, the “Multimodel rehabilitation program” described in the cohort study of Pietilä-Holmner et al. [33] seemed to change coping strategies. Employees who participated in the program scored higher in the coping strategy engagement and the coping strategy pain willingness and had a lower rate of sick leave 1 year after the intervention.

### Consultation with OPs, IPs and a patient representative

Two OPs, two IPs and a patient representative were consulted to give feedback on the findings of this scoping review. The OPs and IPs recognized interventions or components of the interventions and had experience with recommending them to employees. The patient representative recognized components of interventions in the interventions she had followed.

We asked the OPs and IPs specifically about their experience with interventions on changing motivation and optimism/pessimism, because on these factors no interventions had been identified in this scoping review. They were not aware of interventions on these factors either. However, they indicated that they would try to influence some cognitions and perceptions of employees, for example motivation and self-efficacy, by themselves during their consultations.

For choosing an intervention that they would recommend to employees in daily practice, they would, however, not only look at the effectiveness of the intervention. They also considered it very important to look at the type of client (e.g. level of education) and the disease or disorder he or she has, for choosing the right intervention. Some physicians mentioned the importance of deciding together with the employee which intervention is the best fit for the employee. The patient representative emphasized that her preference for one intervention above another is partially based on how much expertise the providers have with the interventions. Because in the Netherlands the employer has to pay for the intervention, the costs of the intervention, the amount of money the employer wants to invest in the employee and the reimbursement policies of insurance companies are all important for determining whether interventions are recommended or not. Some OPs and IPs mentioned that most of the time it is not one person-related factor, but multiple negative cognitions and perceptions that are present in employees, which could make it important to combine interventions or components of interventions.

## Discussion

In this scoping review, we identified 29 studies, of which 23 with a moderate risk of bias, that studied interventions aimed at changing at least one of ten cognitions and perceptions in order to change work participation. The interventions included in the study mainly focused on changing recovery and RTW expectations, self-efficacy, feelings of control, perceived health, fear-avoidance beliefs, perceived work-relatedness of the health problem, coping strategies and catastrophizing. We found no interventions on changing motivation or on optimism/pessimism.

From the results of this review, we can conclude that four interventions were effective in changing cognitions and perceptions and work participation, and can be recommended to employees by OHPs: The “Stimulating health participation and relapse prevention at work” intervention described by Arends et al. [27], the “Cognitive behavioral group intervention on work-anxiety” described by Muschalla et al. [15], the “Combined cognitive behavioral pain competence and depression prevention training” described by Hampel et al. [12] and “Classification-based cognitive functional therapy” described by Vibe Fersum et al. [26]. These interventions were effective in changing work participation by changing coping [15, 27], perceived work-relatedness [15], self-efficacy [12] or fear-avoidance beliefs [26]. Two of the four interventions [26, 27] involved individual sessions with employees and two interventions [12, 15] involved group sessions.

The four interventions that were effective in changing cognitions and perceptions and in increasing work participation had only one main provider, and this was an occupational physician [27], psychological therapist [12, 15] or physiotherapist [26]. This is in contrast to a review of Hoefsmit et al. [45] in which they conclude that it is especially multidisciplinary interventions in which multiple professionals are involved, that seem effective in increasing work participation. However, as we looked at the effectiveness of the intervention on work participation and on one specific cognition or perception, it might not be surprising that it was especially mono-disciplinary interventions that seem to be effective. Besides, many multidisciplinary interventions that were found in this scoping review were studied in cohort studies instead of RCTs. From these studies we cannot conclude whether the interventions are effective or not because they do not compare the change in the cognitions and perceptions and work participation between an intervention and a control group, while many of these interventions seemed to change cognitions and perceptions and work participation over time. An example of this is the intervention of Asih et al. [40], which changed coping profiles over time, which, in turn, had a positive effect on the work retention rate. Therefore, it is possible that more of the described interventions in this scoping review are effective, but that the effectiveness has just not been studied in RCTs yet.

Some of the interventions found in this scoping review which were specifically aimed at one person-related factor also had effects on other person-related factors. For example, the intervention of Muschalla et al. [15] on developing and training coping strategies also had an effect on perceived work-relatedness. This could indicate that some of the cognitions and perceptions are related to other cognitions and perceptions. This is in line with a study by Petrie and Weinmann [46] and a study of Woodhouse et al. [47], which describe that illness perceptions, such as beliefs about the cause of the illness, can influence coping strategies. It might be that changing one cognition or perception could have an effect on another cognition or perception as well.

For certain cognitions and perceptions, no interventions were found at all. This was the case for the factors motivation and optimism/pessimism. The OPs and IPs we approached did not know interventions specifically aimed at these cognitions and perceptions either. However, they did mention that they sometimes try to influence the cognitions and perceptions (such as motivation) of the employees during their consultations without implementing a specific intervention. This is in line with the results of two studies of Müssener et al. [48, 49] in which patients said that encounters with physicians could affect different cognitions and perceptions, such as motivation. So, it is possible that some cognitions and perceptions could also be affected during consultations.

### Strengths and limitations

This review provides an overview of interventions aimed at changing cognitions and perceptions and work participation. OPs, IPs and other OHPs can use this overview to get an indication of which intervention they should recommend in order to increase work participation in employees with chronic health problems. We followed all the steps of the framework of Arksey and O’Malley [7] for conducting this scoping review including the essential last step as described by Levac et al. [5] in which we consulted important stakeholders (e.g. OPs, IPs and a patient representative). This provided additional information into the factors that we should keep in mind when putting these findings into practice, such as the costs and the target audience of the intervention.

A limitation of this review might be that some interventions are tested on specific groups, for example on employees with depression [13, 35]. It is possible that cognitions and perceptions are different between groups. For example, fear-avoidance beliefs can be a factor that is more often present in people who experience pain than in people with other health problems. In addition, components of some interventions are not applicable to employees with other health problems. For example, in the interventions described by Harris et al. [23] participants get homework assignments with exposure to pain-provoking physical activity. This component of the intervention is not applicable for employees who do not have pain when they are physically active. Therefore, the question remains how generalizable the results of studies on interventions tested on specific groups are to a broader population or employees with other health problems. Another limitation is that although the results show effectiveness of some interventions on changing cognitions and perceptions and changing work participation, it remains unclear which part or component of the intervention does have an actual effect on the person-related factor. This is especially the case for multidisciplinary interventions that focus on many different aspects.

### Implications for practice and future research

This review provides an overview of interventions that focus on changing cognitions and perceptions and work participation. OHPs may use the overview of interventions to help employees with chronic health problems to increase work participation.

Many of the identified interventions were not proven effective. Therefore, more studies, and especially more RCTs with a low risk of bias, are needed to study how hindering cognitions and perceptions can be limited and positive cognitions and perceptions fostered. In addition feasibility studies are needed to assess the practicality of the different interventions. Because many of the interventions included in the review are multidisciplinary interventions that focus on many different aspects and are also tested on different groups of employees, it is also important to study which component of the interventions actually helps for which group of employees. According to the consulted stakeholders the expertise of the intervention provider, the type of client (e.g. level of education) and the disease or disorder he or she has are very important to consider when recommending interventions. Results of research assessing which intervention components work for whom, may contribute to the development of more effective and efficient interventions to increase work participation. Finally, research is needed to determine whether these newly developed interventions actually could improve work participation and whether they are cost-effective, because costs are a very important aspect for OHPs in determining whether they should recommend an intervention according to the consulted stakeholders.

## Conclusion

In conclusion, 29 studies were found which described interventions that focused on cognitions and perceptions and were aimed at increasing work participation. Four of these interventions [12, 15, 26, 27] are proven to be effective in RCTs and could be recommended by OHPs to employees in order to change cognitions and perceptions and increase work participation. However, most studies that were included had a moderate risk of bias, so caution should be used when recommending these interventions towards employees. More RCTs with a low risk of bias are needed to explore which of these and other promising interventions that were studied in other study designs are most effective (generally and in terms of costs). In addition more studies are needed to explore which components work for whom in order to increase the generalizability of the findings.

## Supplementary information

**Additional file 1:** **Table 1**. Ovid MEDLINE search strategy. **Table 2**. Ovid PsycINFO search strategy.

**Additional file 2:** **Table 1**. Details of included studies.

**Additional file 3:** **Table 1**. Risk of bias of randomized controlled trials. **Table 2**. Risk of bias of cohort studies. **Table 3**. Risk of bias of non-randomized experimental studies and studies with a single group pre-test post-test design.

## Data Availability

Not applicable.

## Abbreviations

RTW: Return to work
OHPs: Occupational health professionals
OPs: Occupational physicians
IPs: Insurance physicians
PRISMA-ScR: PRISMA Extension for Scoping Review
RCT: Randomized controlled trial
