# Supplementary material for: Interventions on cognitions and perceptions that influence work participation of employees with chronic health problems: a scoping review
Source: BMC Public Health. 2020 Oct 27;20:1610. doi: 10.1186/s12889-020-09621-5 (PMC7590449; doi:10.1186/s12889-020-09621-5)
Supplement: Supplementary file 1 — Additional file 1: Table 1. Ovid MEDLINE search strategy. Table 2. Ovid PsycINFO search strategy. [file 12889_2020_9621_MOESM1_ESM.pdf]

Additional file 1

Table 1. *Ovid MEDLINE search strategy*

Ovid MEDLINE(R) ALL <1946 to June 12, 2020> Search date: 15 June 2020		
**#**	**Searches**	**Results**
1	exp industry/ or exp work/ or exp employment/	448189
2	manpower.fs,sh.	7447
3	(worka* or worke* or workg* or worki* or workl* or workp* or work capacity or work disabilit* or work abilit* or at work or work exposure or work related or workers or job* or employee or staff or personnel or occupation or occupations or occupational or outdoor work* or day shift* or night shift* or shift work* or vocational rehabilitation or sick leave or absenteeism or sickness absen* or absente* or presente* or "return to work" or vocational reintegration or retirement or pension or employment or unemployed or unemployment or work status or industries or industrial sector or repetitive work).ab,kf,ti.	1962185
4	or/1-3 [work]	2291579
5	exp chronic disease/ or long-term care/ or return to work/	288792
6	(((long term or longterm or chronic*) adj3 (ill or illness or disease? or disorder? or condition? or health or sick* or disabil* or injur* or trauma* or care)) or pain or rtw or "return to work" or (month? adj3 (sick or ill))).ab,kf,ti.	1028871
7	(follow up or chronic).hw.	1157609
8	or/5-7	1989602
9	clinical trial.mp.	708643
10	clinical trial.pt.	523177
11	random:.mp. or tu.xs.	5563273
12	(therap* or treatment? or intervention? or rehabilitation).mp.	8949976
13	or/9-12 [therapy]	10868461
14	motivation/ or catastrophization/ or self concept/ or self efficacy/ or adaptation, psychological/	221232
15	(expectation? or belief? or motivation* or unmotivated or willingness or drive or coping or fear avoidance or kinesiophobia or "locus of control" or pain control or personal control or optimism or optimistic or pessimis* or positive outlook or hopelessness or catastrophizing or catastrophization or negativity or ((negative or catastrophic) adj2 (perception? or thinking or thoughts)) or self concept or self esteem or self efficacy or self confiden* or perceived health or "state of health" or perceived severity or self perce* or blam* or work relatedness or (worker? adj2 interview*)).ab,kf,ti.	529332
16	or/14-15 [factors]	656820
17	(life orientation test or "lot-r" or lot revised or illness perception? questionnaire or ipq or coping strateg* questionnaire or csq or pain coping inventory or fear avoidance beliefs questionnaire or fabq or pain management inventory or avoidance endurance questionnaire or aeq or tampa scale or "health locus of control scale" or hlc or mastery scale or pain catastrophizing scale or self efficacy scale or "rtw-se" or ((general health or health status) adj3 (measur* or report* or rate? or rating)) or (general health and (sf36 or sf 36 or shortform 36 or short form 36))).ab,kf,ti.	19328
18	13 and ((visual analog scale or vas) and general health).ab,kf,ti.	426
19	13 and (((general health or health status) adj3 (measur* or report* or rate? or rating)) or (general health and (sf36 or sf 36 or shortform 36 or short form 36))).ab,kf,ti.	6968
20	or/17-19 [relevant inventories]	19540
21	4 and 20	3257
22	and/4,8,13,16	9968
23	21 or 22	12878
24	limit 23 to yr="2013-current"	5520

Table 2. *Ovid PsycINFO search strategy*

#	Searches	Results
	**Ovid PsycINFO <1806 to October Week 2 2020>**<br>**Search date: 15 June 2020**	
**#**	**Searches**	**Results**
1	exp employment/ or exp personnel/ or occupational exposure/ or industrial accidents/ or occupational health/ or occupational safety/ or work related illnesses/	532931
2	(worka* or worke* or workg* or worki* or workl* or workp* or work capacity or work disabilit* or work abilit* or at work or work exposure or work related or workers or job* or employee or staff or personnel or occupation or occupations or occupational or outdoor work* or day shift* or night shift* or shift work* or vocational rehabilitation or sick leave or absenteeism or sickness absen* or absente* or presente* or "return to work" or vocational reintegration or retirement or pension or employment or unemployed or unemployment or work status or industries or industrial sector or repetitive work).ab,id,ti.	863151
3	or/1-2 [work]	1172287
4	chronic illness/ or "chronicity (disorders)"/ or long term care/	21012
5	(((long term or longterm or chronic*) adj3 (ill or illness or disease? or disorder? or condition? or health or sick* or disabil* or injur* or trauma* or care)) or pain or rtw or "return to work" or (month? adj3 (sick or ill))).ab,id,ti.	155769
6	(follow up or chronic).hw.	32637
7	or/4-6	165693
8	clinical trial.mp.	14982
9	random:.mp.	210548
10	(therap* or treatment? or intervention? or rehabilitation).mp.	1215793
11	or/8-10 [therapy]	1317785
12	motivation/ or catastrophization/ or self concept/ or self esteem/ or self efficacy/ or attribution/ or coping behavior/ or "stress and coping measures"/ or "internal external locus of control"/	209489
13	(expectation? or belief? or motivation* or unmotivated or willingness or drive or coping or fear avoidance or kinesiophobia or "locus of control" or pain control or personal control or optimism or optimistic or pessimis* or positive outlook or hopelessness or catastrophizing or catastrophization or negativity or ((negative or catastrophic) adj2 (perception? or thinking or thoughts)) or self concept or self esteem or self efficacy or self confiden* or perceived health or "state of health" or perceived severity or self perce* or blam* or work relatedness or (worker? adj2 interview*)).ab,id,ti.	586298
14	or/12-13 [factors]	634126
15	(life orientation test or "lot-r" or lot revised or illness perception? questionnaire or ipq or coping strateg* questionnaire or csq or pain coping inventory or fear avoidance beliefs questionnaire or fabq or pain management inventory or avoidance endurance questionnaire or aeq or tampa scale or "health locus of control scale" or hlc or mastery scale or pain catastrophizing scale or self efficacy scale or "rtw-se" or ((general health or health status) adj3 (measur* or report* or rate? or rating)) or (general health and (sf36 or sf 36 or shortform 36 or short form 36))).ab,id,ti,tm.	22920
16	11 and ((visual analog scale or vas) and general health).ab,id,ti,tm.	45
17	11 and (((general health or health status) adj3 (measur* or report* or rate? or rating)) or (general health and (sf36 or sf 36 or shortform 36 or short form 36)) or (mos adj3 "36")).ab,id,ti,tm.	2409
18	or/15-17 [relevant inventories]	23297
19	3 and 18	5798
20	and/3,7,11,14	4232
21	19 or 20	9653
22	limit 21 to yr="2013-current"	4429
