# Supplementary material for: Interventions on cognitions and perceptions that influence work participation of employees with chronic health problems: a scoping review
Source: BMC Public Health. 2020 Oct 27;20:1610. doi: 10.1186/s12889-020-09621-5 (PMC7590449; doi:10.1186/s12889-020-09621-5)
Supplement: Supplementary file 2 — Additional file 2: Table 1. Details of included studies. [file 12889_2020_9621_MOESM2_ESM.pdf]

Additional file 2

Table 1. *Details of included studies*

First author, year, country (continent)	Study design	Population<br>N: Number of subjects<br>A: Age; mean age (SD)<br>G: Gender<br>H: Health status	Description intervention<br>D: Duration or number of sessions<br>S: Individual or group sessions<br>P: Provider(s) of intervention<br>M: Main components	Description control intervention<br>D: Duration or number of sessions<br>S: Individual or group sessions<br>P: Provider(s) of intervention<br>M: Main components	Cognitions and perceptions	Follow-up	Risk of bias	Results
Aasdahl L. et al.<br>2018 (28)<br>Norway (Europe)	Single group pre-test post-test study	N: 168<br>A: 47.0 (8.8)<br>G: 32 males, 136 females<br>H: Musculoskeletal, psychological or general and unspecified diagnoses of the International Classification of Primary Care	**Short inpatient program**<br>D: 4+4 days with 2 weeks at home in-between<br>S: Individual and group sessions<br>P: Coordinators with diverse backgrounds (physical therapy, psychology, exercise physiology, nursing or other)<br>M: - Group discussions based on acceptance and commitment therapy<br>- Psychoeducation on stress<br>- Meetings with coordinators and physicians<br>- Mindfulness sessions<br>- Individual and group based supervised training sessions<br>- Creating a RTW plan<br>- Meeting with employer<br><br>**Long inpatient program**<br>D: 3.5 weeks<br>S: Individual and group sessions<br>P: Coordinators with diverse backgrounds (physical therapy, psychology, exercise physiology, nursing or other)<br>M: - Group discussions based on acceptance and commitment therapy<br>- Psychoeducation	-	- Expectations about length of sick leave (one question: “For how long do you believe you will be sick listed from today?”)	9 months	Moderate	- Expectations about sick leave duration significantly changed after the programs (*p* = .01). 56 (33%) participants improved their expectations, 32 (19%) participants reduced their expectations and 80 (48%) participants did not change their expectations.<br>- At 9 months follow-up, sustainable RTW was achieved by 69 participants (41%) and the median of work participation days was 113. No information on significance of increase of work participation is provided.<br>- A positive change in expectations was associated with sustainable RTW (*p* < .01) and work participation days (*p* < .01).

			- Meetings with coordinators and physicians<br>- Mindfulness sessions<br>- Individual and group based supervised training sessions<br>- Walking to work<br>- Creating a RTW plan<br>- A day with outdoor activities<br>- Network day in which participants bring persons to gain insight in the rehabilitation process<br><br>**Outpatient program**<br>D: Once a week for 6 weeks, each session lasted 2.5 hours<br>S: Group sessions<br>P: Physicians, psychologists, social worker, physiotherapist.<br>M: - Group discussions based on acceptance and commitment therapy<br>- Group discussions on physical activity<br>- Sessions with social worker and acceptance and commitment therapy moderator<br>- Home practice, including mindfulness					
Aasdahl L. et al. 2019 (38)<br>Norway (Europe)	Randomized controlled trial	**Short inpatient program**<br>N: 92<br>A: 45.0 (8.7)<br>G: 21 males, 71 females<br>H: Musculoskeletal, psychological or general and unspecified diagnoses of the International Classification of Primary Care<br><br>**Short outpatient program**<br>N: 76<br>A: 45.1 (9.6)	**Short inpatient program**<br>D: 4+4 days with 2 weeks at home in-between<br>S: Individual and group sessions<br>P: Coordinators with diverse backgrounds (physical therapy, psychology, exercise physiology, nursing or other)<br>M: - Group discussions based on acceptance and commitment therapy<br>- Psychoeducation on stress<br>- Meetings with coordinators and physicians<br>- Mindfulness sessions<br>- Individual and group based supervised training sessions<br>- Creating a RTW plan	**Outpatient program (long and short program are identical)**<br>D: Once a week for 6 weeks, each session lasted 2.5 hours<br>S: Group sessions<br>P: Physicians, psychologists, social worker, physiotherapist.<br>M: - Group discussions based on acceptance and commitment therapy<br>- Group discussions on physical activity<br>- Sessions with social worker and acceptance and commitment therapy moderator	- Fear-avoidance beliefs (Fear-Avoidance Beliefs Questionnaire)	3, 9, 12 months	Moderate	- Fear-avoidance beliefs for work decreased from baseline to 12 months follow-up for the participants in the short inpatient program and the short outpatient program, but there was no significant difference between the trials. Fear-avoidance beliefs for work decreased from baseline to 12 months follow-up for the participants in the long inpatient program and the long outpatient program, but there was no significant difference between the trials.<br>- 74% of the participants with a psychological diagnosis and 63% of the participants with a musculoskeletal diagnosis, reduced their fear-avoidance beliefs for work after 12 months.<br>- Participants that had reduced fear-avoidance beliefs for work at 9 moths had 30 more work days than participants with increased scores in fear-avoidance and 43 more work days than participants with consistently high scores of fear-avoidance beliefs, but

		G: 14 males, 62 females<br>H: Musculoskeletal, psychological or general and unspecified diagnoses of the International Classification of Primary Care<br><br>**Long inpatient program**<br>N: 86<br>A: 46.3 (8.7)<br>G: 16 males, 70 females<br>H: Musculoskeletal, psychological or general and unspecified diagnoses of the International Classification of Primary Care<br><br>**Long outpatient program**<br>N: 80<br>A: 45.2 (10.4)<br>G: 19 males, 61 females<br>H: Musculoskeletal, psychological or general and unspecified diagnoses of the International Classification of Primary Care	- Meeting with employer<br><br>**Long inpatient program**<br>D: 3.5 weeks<br>S: Individual and group sessions<br>P: Coordinators with diverse backgrounds (physical therapy, psychology, exercise physiology, nursing or other)<br>M: - Group discussions based on acceptance and commitment therapy<br>- Psychoeducation<br>- Meetings with coordinators and physicians<br>- Mindfulness sessions<br>- Individual and group based supervised training sessions<br>- Walking to work<br>- Creating a RTW plan<br>- A day with outdoor activities<br>- Network day in which participants bring persons to gain insight in the rehabilitation process	- Home practice, including mindfulness				23 less work days than participants with consistently low scores of fear-avoidance beliefs for work.
Adams H. et al. 2017 (33) Canada (North-America)	Cohort study	N: 80<br>A: Males: 46.7 (9.5), females: 45.7 (8.3)<br>G: 26 males, 54 females<br>H: Major depressive disorder	**Risk-targeted activity-reintegration intervention/Progressive goal attainment program:**<br>D: 1 session a week during 10 weeks<br>S: Individual sessions<br>P: Occupational therapist	-	- Catastrophizing (Symptom Catastrophizing Scale)	1 month	Low	- Catastrophizing scores reduced form 10.6 (2.7) to 6.2 (3.3) after the intervention (*p* < .001).<br>- At 1 month follow-up, 21 participants (26%) had returned to work full-time, 3 participants (4%) had returned part-time, 36 participants (45%) were enrolled in a RTW program, and 20 participants (25%) remained absent. No information on significance of increase of work participation is provided.

			M: - Goal setting<br>- Activity planning<br>- Learning techniques targeting disability beliefs<br>- Thought monitoring to target catastrophic thinking<br>- Exposing techniques to facilitate re-engagement in avoided activities<br>- Problem solving challenges to resume occupational activities					- Reductions in catastrophizing predicted occupational re-engagement at the follow-up ($p = .01$).
Arends I. et al.<br>2014 (43)<br>Netherlands (Europe)	Randomized controlled trial	**Stimulating healthy participation and relapse prevention at work (SHARP-at work) intervention:**<br>N: 80<br>A: 41.3 (9.4)<br>G: 27 males, 53 females<br>H: Common mental disorders<br><br>**CAU:**<br>N: 78<br>A: 43.3 (9.8)<br>G: 38 males, 40 females<br>H: Common mental disorders	**SHARP-at work intervention:**<br>D: 2-5 consultations of 30 minutes within 3 months after RTW<br>S: Individual sessions<br>P: Occupational physician<br>M: Problem solving process consisting of:<br>- Inventory of problems at work<br>- Brainstorming on solutions<br>- Note solutions and support needed<br>- Discussion about solutions and making an action plan<br>- Evaluation of action plan	**CAU:**<br>According to guideline on: “Management of mental health problems of workers by occupational physicians”	- Coping (Utrecht Coping List)	3, 6, 12 months	Moderate	- No significant differences in using the three coping strategies (problem focused, emotional and distraction) at all follow-up measurements between the CAU and the SHARP group, except for the coping strategy distraction which was more used by the SHARP group at 3 months follow-up ($p < .05$).<br>- The SHARP group had a lower incidence of recurrent sickness absence than the CAU group at 3 months (11% vs 22%), 6 months (21% vs 39%) and at 12 months (34% vs 47%), ($p < .05$). No information on within-group difference is provided.<br>- Time to recurrent sickness absence was longer in the SHARP group (median of 365 days) as compared to the CAU group (median of 253 days), ($p < .05$).
Asih S. et al. 2015 (44)<br>U.S.A. (North-America)	Cohort study	N: 716<br>A: Adaptive coper: 46.0 (10.8), interpersonally distressed: 45.2 (9.5), dysfunctional: 44.9 (10.1), anomalous: 49.0 (11.2)<br>G: 439 males, 277 females<br>H: Chronic disabling occupational musculoskeletal disorders	**Functional restoration program (FRP):**<br>D: 160 hours, during 4-6 weeks<br>S: Individual and group sessions<br>P: Physical therapist, occupational therapist<br>M: - Evaluations aimed to individually tailored treatment targets<br>- Mobility, strength and fitness training<br>- Cognitive behavior therapy<br>- Counseling<br>- Stress management training	-	- Coping (Coping profiles from Multidimensional Pain Inventory (MPI) questionnaire)	12 months	Moderate	- Coping profiles changed after the FRP ($p < .001$). After FRP, there was an increase in the overall number of patients who became Adaptive coper (AC) (from 32.8% to 47.9%) or Anomalous (from 6.8% to 22.5%) and a decrease in Dysfunctional copers (DYS) (from 39.4% to 14.9%) or Interpersonally distressed (ID) (from 21% to 14.7%).<br>- There was a significant association between coping profiles at discharge and work retention. The DYS had a work retention rate of 64.4% compared to 85.1% in the Anomalous group, 82.2% in the AC group and 74.5% in the ID group ($p = .009$).<br>- Of the 457 participants who completed the program, 369 returned to work. Information about significance was not provided.

			- Coping skills training<br>- Fear-avoidance beliefs training<br>- Patient education about overcoming disability and vocational reintegration<br>- Medical supervision including medication management, interventions to improve function, and an assessment of remaining surgical options					- There was no association between coping profiles at discharge and RTW rate.
Chu M. C. et al. 2015 (17) China (Asia)	Cohort study	N: 142<br>A: 42.0, range 21-62<br>G: 57 males, 85 females<br>H: Chronic non-cancer pain	**Comprehensive outpatient pain engagement (COPE) program:**<br>D: 100 hours during 14 days<br>S: Group sessions<br>P: Psychologist, physiotherapist, occupational therapist, pain nurse, hospital chaplain, medical social worker<br>M: - Education about pain pathophysiology<br>- Behavioral training<br>- Pacing, relaxation, strengthening and stretching exercises<br>- Thought management<br>- Communication<br>- Activity planning<br>- Appropriate use of medication	-	- Catastrophizing (Pain Catastrophizing Scale)<br>- Self-efficacy (Patient Self Efficacy Questionnaire)<br>- Perceived health (36-item Short-Form Health Survey)	12 months	Moderate	- Catastrophizing decreased one year after the program, from a mean of 34.1 (11.1) to 25.8 (14.1) ($p < .001$).<br>- Self-efficacy increased one year after the program, from 23.2 (11.0) to 30.6 (13.9) ($p < .001$).<br>- Perceived health improved one year after the program from 21.4 (18.1) to 36.7 (23.0) ($p = .03$).<br>- Work status improved one year after the program, with 35% of the participants working after the program, as compared to 17% before the program ($p = .0002$).
Fauser D. et al. 2019 (24) Germany (Europe)	Randomized controlled trial	**Conventional rehabilitation plus additional work-related modules:**<br>N: 229<br>A: 50.8 (7.1)<br>G: 66 males, 163 females<br>H: Cancer<br><br>**Conventional medical rehabilitation**<br>N: 255<br>A: 50.3 (7.9)<br>G: 94 males, 161 females	**Conventional medical rehabilitation plus additional work-related modules:**<br>D: 100 hours<br>S: Individual and group sessions<br>P: Physician, psychologist, psychotherapist, occupational therapist, physiotherapist, social worker<br>M: - Exercise therapy<br>- Physiotherapy<br>- Social counseling<br>- Occupational therapy<br>- Nutritional advice<br>- Psychological seminars and counseling	**Conventional medical rehabilitation:**<br>D: 60-75 hours during 3 weeks<br>S: -<br>P: -<br>M: - Exercise therapy<br>- Physiotherapy<br>- Social counseling<br>- Occupational therapy<br>- Nutritional advice<br>- Psychological seminars and counseling<br>- Medical treatment and counseling	- Perceived health (European Organization for Research and Treatment of Cancer Quality of Life Questionnaire; EORTC QLQ-C30)<br>- Coping (Freiburg Questionnaire of Coping with Illness)	12 months	Moderate	- For perceived health and coping there were no significant differences between the intervention and control group 1 year after completing the programs. No information on significance of within-group difference is provided.<br>- After 1 year 28.5% of the intervention group and 25.3% of the control group had still not returned to work. No information on significance of within-group difference is provided. There were no significant differences in time until RTW between the intervention and control group.

		H: Cancer	- Medical treatment and counseling<br>- Work-related diagnostic evaluation<br>- Intensive social counseling<br>- Work-related psychosocial groups<br>- Work-related functional capacity training					
Gagnon C. M. et al. 2013 (31) U.S.A. (North-America)	Cohort study	N: 101<br>A: 43.5 (8.2)<br>G: 64 males, 37 females<br>H: Chronic pain	**Interdisciplinary pain management program:**<br>D: 8 hours for 5 days during 4 weeks<br>S: Individual and group sessions<br>P: Physicians<br>M: - Vocational counseling<br>- Psychological treatment<br>- Occupational therapy<br>- Physical therapy<br>- Biofeedback/relaxation training<br>- Aerobic conditioning<br>- Physician appointments<br>- Pool therapy<br>- Education<br>- Feldenkrais movement therapy	-	- Catastrophizing (Pain Catastrophizing Scale)	At discharge from program	High	- Catastrophizing decreased at discharge from the interdisciplinary pain management program from a mean score of approximately 28 to 24 (*p* = .033).<br>- From the program completers, 49% were working, whereas 12% were working at the start. No information on significance is provided.<br>- A greater percentage of the program completers was working as compared to the non-completers (49% vs approximately 9%), (*p* = .005).
Granviken F. et al. 2015 (39) Norway (Europe)	Randomized controlled trial	**Home exercise intervention:**<br>N: 23<br>A: 48.2 (9.8)<br>G: 12 males, 11 females<br>H: Subacromial impingement<br><br>**Supervised exercise intervention:**<br>N: 23<br>A: 47.6 (10.0)<br>G: 12 males, 11 females<br>H: Subacromial impingement	**Supervised exercise intervention:**<br>D: 10 supervised sessions and exercises at home for 6 weeks<br>S: Individual sessions<br>P: Physiotherapist<br>M: -Theory lesson on anatomy and rehabilitation<br>- Supervised exercise therapy focused on re-establishing normal shoulder movement patterns<br>- Home exercises	**Home exercise intervention:**<br>D: 1 supervised session and exercises at home for 6 weeks<br>S: Individual sessions<br>P: Physiotherapist<br>M: -Theory lesson on anatomy and rehabilitation<br>- A session with physiotherapist to set up a tailored home-exercise program focused on re-establishing normal shoulder movement patterns	- Fear-avoidance beliefs (Fear-Avoidance Beliefs Questionnaire)	6, 26 weeks	Moderate	- Fear-avoidance beliefs decreased after 6 weeks with -3.2 (5.5) in the home exercise group and -3.1 (7.8) in the supervised exercise group. No information on significance of within-group difference is provided. There were no significant differences between the groups.<br>- At 6 weeks, 7 of the 21 participants in the home exercise group were on sick leave and in the supervised exercise 10 of the 23 participants were on sick leave. At 26 weeks, 4 of the 18 participants in the home exercise group and 3 of the 21 participants in the supervised exercise group were on sick leave. No information on significance of within-group difference is provided. There were no significant differences between the groups.

Haiduk P. et al. 2017 (27) Switzerland (Europe)	Cohort study	N: 59<br>A: 40.3 (12.3)<br>G: 10 males, 49 females<br>H: Chronic neck pain	**The 4 interdisciplinary pain program:**<br>D: 24.5-27.5 hours per week during 4 weeks<br>S: Individual and group sessions<br>P: Clinical neuropsychologists, physicians, physiotherapists, occupational therapists, Qi Gong instructors, creative therapists<br>M: - Physiotherapy<br>- Strength and endurance training<br>- Occupational therapy<br>- Cognitive behavioral and coping therapy<br>- Relaxation<br>- Music and painting therapy<br>- Tai Chi and Qigong	-	- Catastrophizing (Coping Strategies Questionnaire)<br>- Perceived health (36-item Short-Form Health Survey)	6, 60 months	Moderate	- Catastrophizing improved from a mean of 57.5 (18.2) at entry to 63.7 (20.0) at 6 months ($p = .03$) and to 76.2 (23.5) at the 60 months ($p < .001$) follow-up after the 4 interdisciplinary pain program.<br>- Perceived health did not change at 6 months, but improved from 52.6 (17.3) at entry to 60.5 (20.8) after 60 months ($p = .01$).<br>- Median working capacity increased from 0 hours a week at entry, to 9 hours a week at 6 months to 30 hours a week after 60 months. No information on significance is provided.
Hampel P. et al. 2019 (16) Germany (Europe)	Randomized controlled trial	**Combined cognitive-behavioral pain competence and depression prevention training**<br>N: 295<br>A: 53.3 (6.0)<br>G: 53 males, 242 females<br>H: Chronic low back pain<br><br>**Pain competence training**<br>N: 288<br>A: 53.3 (6.1)<br>G: 53 males, 235 females<br>H: Chronic low back pain	**Combined cognitive-behavioral pain competence and depression prevention training**<br>D: 8 sessions of 100 minutes in 3-4 weeks<br>S: Group sessions<br>P: Psychotherapist<br>M: - Standard inpatient multidisciplinary rehabilitation<br>- Four sessions of 75 minutes of pain competence training in order to treat pain-related fear-avoidance beliefs and improve stress and pain management to promote self-management and self-efficacy expectations<br>- Four sessions of 75 minutes of depression prevention training, including enhancement of the activity level, cognitive restructuring, social skills training, discussing cognitions and behaviors, and practicing coping strategies.<br>- Eight unguided group workshops of 25 minutes to	**Pain competence training**<br>D: 4 sessions of 100 minutes in 3-4 weeks<br>S: Group sessions<br>P: Psychotherapist<br>M: - Standard multidisciplinary rehabilitation<br>- Four sessions of 75 minutes of pain competence training in order to treat pain-related fear-avoidance beliefs and improve stress and pain management to promote self-management and self-efficacy expectations<br>- Four unguided group workshops of 25 minutes to complete homework assignments	- Pain self-efficacy (pain self-efficacy questionnaire)	6,12 months	Moderate	- Pain self-efficacy increased over time in the intervention group in comparing with the control group ($p = .016$), but only for participants with high levels of depressive symptoms.<br>- Pain-related days of sick leave significantly decreased in the intervention group ($p < .001$), but not in the control group.<br>- After 12 months significantly more participants were employed in the intervention group in comparing to the control group ($p < .017$).

			complete homework assignments					
Harris A. et al. 2017 (40)<br>Norway (Europe)	Randomized controlled trial	**Group physical exercise**<br>N: 60<br>A: 44.2 (10.6)<br>G: 32 males, 28 females<br>H: Non-specific low back pain<br><br>**Group cognitive behavioral therapy**<br>N: 55<br>A: 45.5 (9.1)<br>G: 31 males, 24 females<br>H: Non-specific low back pain<br><br>**Brief intervention**<br>N: 99<br>A: 44.8 (9.7)<br>G: 43 males, 56 females<br>H: Non-specific low back pain	**Group physical exercise (Group PE):**<br>D: 3 sessions of 90 minutes a week for 3 months<br>S: Group sessions<br>P: Physiotherapist, psychologist (optional), medical doctor (optional)<br>M: - Brief intervention<br>- Physical exercises adapted to the individual needs<br>- Strength and endurance training<br>- Relaxation<br>- Exposure to physical activity that was perceived as harmful<br>- Two sessions about coping, chronic pain and ergonomics (optional)<br><br>**Group cognitive behavioral therapy (Group CBT):**<br>D: 7 sessions of 90 minutes in 3 months<br>S: Group sessions<br>P: Psychiatrist<br>M: - Brief intervention<br>- Homework consisting of exposure to pain-provoking physical activity<br>- Group discussions about homework and experienced problems in order to change dysfunctional thoughts	**Brief intervention:**<br>D: 2 sessions of 2-4 hours over 5 days, 2 booster sessions (optional)<br>S: Individual sessions<br>P: Specialist in physical medicine and rehabilitation (first session), physiotherapist (second session)<br>M: - Physical examination including diagnostic clarification, reassurance about normal findings, communication about harmlessness of back pain, encouragement of physical activity<br>- Follow-up session with an educational part for strengthening the message given in the medical examination and a behavioral part for turning new insights into practical action	- Coping (Utrecht Coping List)<br>- Fear-avoidance beliefs (Fear-Avoidance Beliefs Questionnaire)	3, 6, 12 months	Moderate	- Coping improved from 3.02 (0.20) at baseline to 3.06 (0.31) at 12 months in the brief intervention group, from 3.06 (0.31) to 3.10 (0.30) for the group CBT and from 3.01 (0.30) to 3.12 (0.30) for the group PE (p = .005), but there was no significant difference between the interventions.<br>- Fear-avoidance beliefs for work decreased from 22.38 (10.7) at baseline to 17.6 (12.92) at 12 months in the brief intervention, from 24.48 (8.83) to 19.31 (11.76) for the group CBT and from 26.03 (9.07) to 18.84 (11.59) for the group PE (p < .001), but there was no significant difference between the interventions.<br>- 60% of the participants in the brief intervention group increased work participation in comparing to 54.6% in the group CBT and 51.7% in the group PE. No information on significance of within-group difference is provided. RTW at 12 months follow-up did not differ between the groups.
Hees H. L. et al. 2013 (12)<br>Netherlands (Europe)	Randomized controlled trial	**Treatment as usual + Occupational therapy (TAU + OT)**<br>N: 78<br>A: 43.8 (9.0)<br>G: 41 males, 37 females<br>H: Major depressive disorder	**TAU + OT:**<br>D: 18 sessions<br>S: Individual sessions, group sessions, one session with employer<br>P: Two occupational therapists<br>M: - Problem clarification including an intake about patients current work situation and their problem areas	**TAU:**<br>D: -<br>S: Individual sessions<br>P: Psychiatrist specialized in depression<br>M: - Treatment in outpatient clinic<br>- Psychoeducation<br>- Supportive therapy<br>- Cognitive behavioral interventions	- Coping (Utrecht Coping List)<br>- Work-related self-efficacy (Expectations regarding work resumption questionnaire)	6, 12, 18 months	Low	- Active problem solving coping improved from a mean of 16.6 (3.8) to 17.9 (3.7) at 18 months follow-up (p < .001), passive reaction coping reduced from 15.8 (4.4) to 13.1 (3.3), (p < .001), avoidance coping decreased from 17.2 (3.2) to 16.9 (3.3), (p = .05) in the intervention group. There were no group differences.<br>- Self-efficacy improved from 3.4 (1.1) to 4.2 (1.0) at 18 months (p < .001) in the intervention group. There were no group differences.<br>- There was a significant decrease in hours of absenteeism, from 22.7 (10.0) to 10.4 (12.5) at 18

		**Treatment as usual (TAU)**<br>N: 39<br>A: 41.5 (9.6)<br>G: 16 males, 23 females<br>H: Major depressive disorder	- Group sessions where the Quality of Work model (model about factors that affect work performance) is discussed<br>- Making a work-reintegration plan<br>- Individual sessions where therapist relates occurring work stressors to patient's ineffective coping-pattern<br>- A meeting with the employer about work-related difficulties<br>- Follow-up session to discuss potential problems during the work resumption process	- Pharmacotherapy (optional)<br>- Day or inpatient treatment (optional)				months ($p < .001$) in the intervention group. There were no group differences.<br>- Median number of days until partial RTW was 80 (42-172) and 361 (193-653) for full RTW for the intervention group. There were no group differences.<br>- In the intervention group, 92% of participants achieved at least partial RTW and 66% achieved full RTW during the study period of 18 months.
Hutting N. et al. 2015 (13) Netherlands (Europe)	Randomized controlled trial	**Self-management intervention:**<br>N: 64<br>A: 45.0 (11.2)<br>G: 11 males, 53 females<br>H: Chronic non-specific complaints of the arm, neck or shoulder (CANS)<br><br>**CAU:**<br>N: 53<br>A: 47.7 (10.5)<br>G: 17 males, 36 females<br>H: Chronic non-specific CANS	**Self-management intervention:**<br>D: 6 weekly sessions of 2.5 hours<br>S: Group sessions<br>P: Moderator<br>M: - Making and discussing action plans<br>- Setting targets in terms of behavior<br>- eHealth module about training, self-management and CANS	**CAU:**<br>All CAU and information available within and outside the organization of the participant	- Pain catastrophizing (Pain Catastrophizing Scale)<br>- Self-efficacy (Dutch Adaptation of the General Self-Efficacy Scale)<br>- Self-efficacy at work (Self-Efficacy at Work Scale)	3, 6, 12 months	Moderate	- Pain catastrophizing decreased from 10.42 at baseline to 9.25 at 12 months follow-up for the intervention group. No information on significance of within-group difference is provided. There was no difference between the groups.<br>- General self-efficacy increased from 31.16 at baseline to 32.91 at 12 months follow-up for the intervention group and self-efficacy at work from 8.62 to 13.58. No information on significance of within-group difference is provided. There was no difference between the groups.<br>- Days absent from work in the past month changed from 1.63 at baseline to 3.42 at 12 months. No information on significance of within-group difference is provided. There was no difference between the groups.
Jensen A. G. C. 2013 (20) Denmark (Europe)	Non-randomized experimental study	**Intervention group:**<br>N: 118<br>A: 34, range 18-63<br>G: 15 males, 103 females<br>H: Mental illness, musculoskeletal illness, mental and musculoskeletal illness<br><br>**Reference group:**	**RTW intervention:**<br>D: Max. one year<br>S: Individual and group sessions<br>P: Social worker, experienced exercise instructor, therapist, rheumatologist<br>M: - An individually tailored rehabilitation plan based on results from a Work Disability Diagnosis interview	-	- Perceived health (36-item Short-Form Health Survey)<br>- General self-efficacy (Generalized Self-Efficacy Scale)	12, 24 months	Low	- Perceived health did not change after the intervention.<br>- Self-efficacy did not change after the intervention.<br>- At two-year follow-up, 64% of the intervention group returned to work in comparing to 48% in the reference group. The odds for RTW were higher in the intervention group at two-year follow-up ($p < .05$), but there was no difference at one-year follow-up.<br>- Mean duration of sick leave at one-year follow-up was significantly lower in the intervention group (28.9 weeks) as compared to the reference group (34.0 weeks) at one-year follow-up ($p < .05$), but there was no difference at two-year follow-up.

		N: 86<br>A: Comparable to intervention group.<br>G: Comparable to intervention group.<br>H: Comparable to intervention group.	- Physical exercises with natural movements of the body (optional)<br>- Ergonomic course with personal guidance at work (optional)<br>- A discussion at the work place about a RTW plan (optional)<br>- Consultation with a rheumatologist for diagnostics and/or treatment (optional)<br>- Cognitive therapy (optional)					- Decline in self-efficacy was predictive for less chance to RTW at the one-year follow-up (*p* < .05), when adjusted for sex and age, but not at two-year follow-up.<br>- Increase in perceived health was predictive for RTW at one-year follow-up (*p* < .05) and at two-year follow-up (*p* < .05) when adjusted for sex and age.
Leensen M. C. J. et al. 2017 (18) Netherlands (Europe)	Single group pre-test post-test study	N: 93<br>A: 47.9 (7.4)<br>G: 9 males, 84 females<br>H: Cancer	**Multidisciplinary rehabilitation program:**<br>D: 2 times a week during 12 weeks<br>S: Individual sessions<br>P: Physiotherapist, oncological occupational physician<br>M: - Supervised interval and resistance exercises<br>- Counselling sessions with advice on gradual work resumption	-	- Self-efficacy (Self-Efficacy Scale)	6, 12, 18 months	Moderate	- Self-efficacy increased at 18 months from a mean of 3.7 (0.8) to 4.2 (0.6) (*p* < .001), but not at 6 months follow-up.<br>- Rate of RTW increased to 59% at 6 months follow-up, 86% at 12 months follow-up and 83% at 18 months follow-up (*p* < .001). The median number of days to RTW was 292.
Marchand G. H. et al. 2015 (41) Norway (Europe)	Randomized controlled trial	**Work-focused intervention:**<br>N: 201<br>A: 40.1 (9.7)<br>G: 111 males, 90 females<br>H: Neck and/or back pain<br><br>**Brief intervention and Multidisciplinary intervention:**<br>N: 197<br>A: 41.1 (10.0)<br>G: 101 males, 96 females<br>H: Neck and/or back pain	**Work-focused intervention:**<br>D: 5-6 days, during 3 weeks<br>S: Individual and group sessions<br>P: Physiotherapist, case-worker<br>M: - Talking with caseworker about work histories, family lives, obstacles to RTW<br>- Contact between caseworker and employer about possible modification at work<br>- Creating a RTW schedule<br>- Contact between caseworker and municipal social service (optional)<br>- Assistance in meeting employer (optional)	**Brief intervention at Oslo University hospital:**<br>D: 3 weeks<br>S: Individual sessions<br>P: Physiotherapist, medical specialist<br>M: - A diagnostic clarification<br>- Session with a physiotherapist consisting of advice in activities, encouragement for exercise<br>- One clarifying session with a medical specialist<br><br>**Multidisciplinary intervention at St. Olav's hospital:**<br>D: 3 weeks<br>S: Individual and group sessions	- Fear-avoidance beliefs (Fear-Avoidance Beliefs Questionnaire, the work subscale).	4, 12 months	Moderate	- Fear-avoidance beliefs for work improved in 26% of the patients in the intervention group and in 20% of the patients in the control group, but no information on significance of within-group difference is provided. There were no significant differences between the groups.<br>- Improvement in fear-avoidance beliefs for work was a positive predictor for RTW (*p* = .023). The odds for RTW increased to 4.0 (*p* = .015) for the group with improved fear-avoidance beliefs for work scores.<br>- Participation in the work-focused intervention was not a significant predictor for RTW within 12 months.

				P: Physiotherapist, medical specialist, social worker<br>M: - Cognitive behavioral therapy<br>- Exercise				
Muschalla B. et al. 2016 (14) Germany (Europe)	Randomized controlled trial	**Cognitive behavioral group intervention on work-anxiety (WAG intervention):**<br>N: 177<br>A: 48.9 (8.7)<br>G: 81 males, 96 females<br>H: Orthopedic disorders, cardiologic disorders, neurological disorders<br><br>**Recreational group (RG) intervention:**<br>N: 168<br>A: 51.4 (8.0)<br>G: 86 males, 82 females<br>H: Orthopedic disorders, cardiologic disorders, neurological disorders	**WAG intervention:**<br>D: Sessions of 90 minutes, 2 times a week for 3 weeks<br>S: Group sessions<br>P: Physician specialized in psychiatry, psychological behavior therapist<br>M: - Developing and training individual cognitive and behavioral strategies to cope with work-anxiety<br>- Situation and behavior analysis<br>- Problem solving<br>- Guided discovery questions<br>- Homework assignments	**RG intervention:**<br>D: Sessions of 90 minutes, 2 times a week for 3 weeks<br>S: Group sessions<br>P: Physician specialized in psychiatry, psychological behavior therapist<br>M: - Unspecific recreational activities such as painting, cooking, playing games<br>- Situation and behavior analysis<br>- Problem solving<br>- Guided discovery questions<br>- Homework assignments	- Work-related coping (Job Coping and Return Intention inventory)<br>- Work-related self-efficacy (Job Coping and Return Intention inventory)<br>- Internal control perception concerning RTW (Job Coping and Return Intention inventory)<br>- Relation between work and health problems (one question: "To which degree are your health problems caused or forced by your (last) work on a scale from 0-100")	6 months	Moderate	- Work-related self-efficacy did not change after both interventions.<br>- Work-related active coping did not change after both interventions. Patients in the WAG showed an increase in the coping strategies self-calming and self-instruction over time as compared to the RG with covariate age (*p* = .025) and with covariate obtaining a workplace (*p* = .037). The increase in the WAG group was from a mean of 3.66 (0.93) to 3.74 (0.77).<br>- Internal and external control perception did not change for both interventions.<br>- Perceived work-relatedness decreased significantly for the WAG as compared to the RG with covariate gender (*p* = .007). The decrease in the WG group was from a mean of 47.69 (30.96) to 45.36 (30.07).<br>- Sick leave duration was 6 months after rehabilitation significantly lower in the WAG group (10.51 weeks) compared to the RG group (15.59 weeks) for patients with work-anxiety only (*p* = .05), but there were no differences between the groups for participants with work-anxiety and general mental disorders or for all the participants.
Pedersen P. et al. 2015 (23) Denmark (Europe)	Randomized controlled trial	**Psychoeducation:**<br>N: 215<br>A: 43.5 (10.0)<br>G: 61 males, 154 females<br>H: Anxiety, depression, other mental illness, stress and burnout, musculoskeletal disorders<br><br>**CAU:**	**Psychoeducation:**<br>D: 6 weekly sessions of 2 hours<br>S: Group sessions<br>P: Psychiatric nurse, psychologist, social worker, physiotherapist, person previously on sick leave due to mental health problems<br>M: - Didactic lectures and group discussions based on problem solving techniques and coping strategies	**CAU:**<br>CAU offered by job centers which typically comprises fitness workout, stress and pain management and gradual RTW. All participants were free to engage in any other treatment.	- Health locus of control (The Multidimensional Health Locus of Control)<br>- Perceived health (one question: "In general, would you say your health is..")	3, 6, 12 months	Moderate	- Internal locus of control changed from 22.0 at baseline, to 23.0 at 3 months follow-up to 24.0 at 6 months follow-up in the intervention group. No information on significance of within-group difference is provided.<br>- Internal locus of control was higher for the intervention group at 3 months follow-up (median 23.0) than for the control group (median 20.0) (*p* < .001) and was higher for the intervention group (median 24.0) than for the control group (median 21.0) at 6 months follow-up (*p* < .001). There were no differences for the other three locus of control variables (chance, doctors, other people).

		N: 215<br>A: 43.9 (9.9)<br>G: 60 males, 155 females<br>H: Anxiety, depression, other mental illness, stress and burnout, musculoskeletal disorders	- Session with relatives to hear about mental health problems and sickness absence					- Perceived health did not differ between the groups at 3 months or 6 months follow-up.<br>- At 3 months more participants in the control group had full RTW than in the intervention group (28% vs 19%), but there were no significant differences at 6 or 12 months.<br>- For first RTW, there were no significant differences between the groups at either time points.
Pietilä-Holmner E. et al. 2020 (26)<br>Sweden (Europe)	Cohort study	N: 234<br>A: 43.6 (10.8)<br>G: 34 males, 200 females<br>H: Chronic musculoskeletal pain	**Multimodal rehabilitation program (MMRP):**<br>D: Sessions of 1.5–3.5 hours a week for 6–10 weeks<br>S: Individual and group sessions<br>P: Physiotherapist, occupational therapist, general practitioner, social worker, psychologist<br>M: - Goalsetting together with the patient<br>- Physical exercise<br>- Relaxation<br>- Training in coping strategies based on cognitive behavioural therapy (CBT)<br>- Education in pain management	-	- Coping (Chronic Pain Acceptance Questionnaire)<br>- Catastrophizing (Pain Catastrophizing Scale)<br>- Perceived health (Visual Analogue Scale)	12 months	Moderate	- Catastrophizing reduced significantly from a median of 21.0 (15.8) at baseline to a median of 19.0 (16.0) at one-year follow-up (p < 0.001).<br>- Perceived health increased significantly from a median of 44.0 (30.0) at baseline to a median of 50.0 (34.0) at one-year follow-up (p < .001).<br>- The coping strategy activity engagement increased from a median of 29.5 (12.5) at baseline to a median of 36.0 (18.0) at follow-up (p < .001). The coping strategy pain willingness increased from 23.0 (11.0) at baseline to 27.0 (11.0) at follow-up (p < .001).<br>- At one-year follow-up, the proportion of patients on sick leave decreased significantly from 39.7% at baseline to 31.6% at the one-year follow-up (p = 0.027).
Rolving N. et al. 2015 (29)<br>Denmark (Europe)	Randomized controlled trial	**Cognitive behavioral therapy (CBT):**<br>N: 59<br>A: 51.4 (9.2)<br>G: 23 males, 36 females<br>H: Degenerative disc disease or spondylolisthesis undergoing LSF<br><br>**CAU:**<br>N: 31<br>A: 47.7 (8.9)<br>G: 16 males, 15 females	**CBT:**<br>D: 6 sessions of 3 hours<br>S: Group sessions<br>P: Psychologist, occupational therapist, physiotherapist, social worker, spine surgeon, previously operated patient<br>M: - Standard course of treatment<br>- Pre- en post-operative sessions<br>- Group discussions about the interaction of cognition and pain perception, coping strategies, pacing principles, ergonomic directions, RTW and details about the surgical procedure	**CAU:**<br>D: 8 weeks<br>S: Individual or group sessions<br>P: Operating surgeon, nurse, physiotherapist, occupational therapist<br>M: - Preoperative information about operation and anesthetics procedure<br>- Medication<br>- Information about postoperative rehabilitation and physical restrictions after surgery<br>- Supervised exercise	- Catastrophizing (Coping Strategies Questionnaire)	3, 6, 12 months	Moderate	- Catastrophizing decreased with -5.0 after 3 months, with -7.5 after 6 months and -5.0 after 1 year in comparing with baseline catastrophizing. No information on significant within-group difference is provided.<br>- Catastrophizing decreased more in the intervention group after 6-months follow-up (-7.5 points) than in the control group (-2.0), (p = .04), but there was no difference in decrease in catastrophizing between the groups at 3 months and one-year follow-up.<br>- At one-year follow-up 42% of the CBT group had resumed work. No information on significant within-group difference is provided. RTW rate and sick leave during the first year did not differ between the groups at one-year follow-up.

		H: Degenerative disc disease or spondylolisthesis undergoing LSF	- Homework about thoughts and feelings in relation to stressful situations, coping strategies, and setting goals					
Ronzi Y. et al. 2017 (42) France (Europe)	Randomized controlled trial	**Functional restoration program (FRP):**<br>N: 49<br>A: 40.0<br>G: 27 males, 22 females<br>H: Non-specific chronic low back pain<br><br>**Ambulatory individual physiotherapy (AIP):**<br>N: 54<br>A: 42.0<br>G: 33 males, 21 females<br>H: Non-specific chronic low back pain<br><br>**Mixed strategy:**<br>N: 56<br>A: 40.0<br>G: 35 males, 21 females<br>H: Non-specific chronic low back pain	**AIP:**<br>D: 1 hour, 3 times a week during 5 weeks<br>S: Individual sessions<br>P: Physiotherapist<br>M: - Active exercises supervised by physiotherapist<br>- 50 minutes of home exercises, three days a week<br><br>**Mixed strategy:**<br>D: 1 hour, 3 times a week + 5 one-day sessions during 5 weeks<br>S: Individual and group sessions<br>P: Physiotherapist, rehabilitation physician, sports therapist, psychologist<br>M: - Ambulatory physiotherapy<br>- Assessment of chronic low back pain perception and discussion of representations and beliefs<br>- Advices on appropriate activities and dietary advices<br>- Relaxation sessions<br>- Meeting with a psychologist	**FRP:**<br>D: 6 hours a day, 5 days a week during 5 weeks<br>S: Group sessions<br>P: Physiotherapist<br>M: - Supervised exercises focused on muscular warm-up and stretching, flexibility, cardio-respiratory, endurance, weightlifting, proprioception, coordination and strengthening	- Fear-avoidance beliefs (Fear-Avoidance Beliefs Questionnaire)	12 months	Moderate	- Fear-avoidance beliefs decreased in the FRP group from 44.0 to 35.5 at 12-months follow-up and in the mixed strategy group from 44.0 to 39.0 ($p < .05$), but not in the AIP group. There were no differences in decreased fear-avoidance beliefs between the groups.<br>- Number of sick leave days decreased in all three treatment groups during 12 months of follow-up from 256.0 to 50.5 in the FRP group, from 209.0 to 47.0 in the mixed strategy group and from 219.0 to 45.0 in the AIP group ($p < .05$). There were no differences in number of sick leave days between the groups.
Salzwedel A. et al. 2020 (19) Germany (Europe)	Cohort study	N: 1262<br>A: 54.2 (7.0)<br>G: 968 males, 294 females<br>H: Cardiovascular diseases	**Standardized comprehensive cardiac rehabilitation (CR) program:**<br>D: 3 to 4 weeks, with 12 sessions per week with a duration of 30-45 minutes and 8 additional sessions<br>S: Individual and group sessions<br>P: Cardiologist, physician and social worker	-	- General self-efficacy expectations **(**Allgemeine Selbstwirksamkeit Kurzskala: short scale for measuring general self-efficacy beliefs)	6 months	Low	- The mean score for general self-efficacy expectations increased after CR from 4.1 (0.7) at admission to 4.1 (0.7) at discharge ($p < 0.001$).<br>- At follow-up 68.5% returned to work, 5.3% had retired, 6.3% had applied for pension, 7.1% were unemployed and 15.1% of the participants were still on sick leave. No information on significance was reported.

			M: - Counseling by a cardiologist<br>- Risk-factor modification strategies (education on nutrition, smoking cessation, physical activity and medication adherence)<br>- Physician-supervised exercise training and sports therapy<br>- Psychosocial interventions<br>- Vocational assessment<br>- Physician and social worker counseling					
Scott W. et al.<br>2014 (30)<br>Canada (North-America)	Single group pre-test post-test study	N: 148<br>A: 36.6 (9.2)<br>G: -<br>H: Whiplash injury	**Multidisciplinary rehabilitation program:**<br>D: 7 weeks<br>S: Individual and group sessions<br>P: Physiotherapist, occupational therapist, psychologist<br>M: - Tailored exercises<br>- Education<br>- Instruction in self-management skills	-	- Pain catastrophizing (Pain Catastrophizing Scale)	12 months	Moderate	- Mean catastrophizing decreased from 22.27 (SD = 10.83) to 13.66 (SD = 11.17) after the multidisciplinary rehabilitation program for participants. No information on significance is provided.<br>- At one-year follow-up, 69.6% of participants had resumed some degree of employment-related activities. No information on significance is provided.<br>- Participants who did not RTW had significantly lower percent reduction on catastrophizing than those who returned to work (*p* = .001). In 72% of the time, individuals who returned to work obtained higher percent change on pain catastrophizing than those who did not return.
Sullivan M. et al.<br>2017 (34)<br>Canada North-America	Cohort study	**Men:**<br>N: 35<br>A: 47.7 (10.6)<br>H: Post-traumatic stress disorder<br><br>**Women:**<br>N: 38<br>A: 44.8 (8.5)<br>H: Post-traumatic stress disorder	**Risk-targeted activity-reintegration intervention/Progressive goal attainment program:**<br>D: 1 session a week during 10 weeks<br>S: Individual sessions<br>P: Occupational therapist<br>M: - Goal setting<br>- Activity planning<br>- Learning techniques targeting disability beliefs<br>- Thought monitoring to target catastrophic thinking<br>- Exposing techniques to facilitate re-engagement in avoided activities<br>- Problem solving challenges to resume occupational activities	-	- Catastrophizing (Symptom Catastrophizing Scale)	1 month	Low	- Catastrophizing decreased with 38% after treatment (*p* < .001).<br>- At 1 month follow-up, 34% of the participants had returned to work full-time and 15% had returned to part-time work. No information on significance is provided.<br>- Participants who returned to work at follow-up, had greater reductions in catastrophizing scores (*p* < .001), than participants who did not return. Change scores on catastrophizing contributed significant variance to the prediction of occupational re-engagement (*p* < .001).

Van Eijk-Hustings Y. et al. 2013 (25) Netherlands (Europe)	Randomized controlled trial	**Multidisciplinary intervention with aftercare (MD):**<br>N: 108<br>A: Started: 41.6 (8.8). Not started: 41.3 (11.0)<br>G: Started: 4 males, 63 females. Not started: 3 males, 38 females.<br>H: Fibromyalgia<br><br>**Aerobic exercise (AE):**<br>N: 47<br>A: Started: 43.9 (7.6). Not started: 39.1 (9.6)<br>G: Started: 0 males, 19 females. Not started: 0 males, 28 females.<br>H: Fibromyalgia<br><br>**CAU:**<br>N: 48<br>A: 42.9 (11.0)<br>G: 1 male, 47 females<br>H: Fibromyalgia	**MD:**<br>D: 1 year. First phase: 12 weeks, 3 days per week, with 2 sessions of 1.5 hour duration per day. Second phase: 5 meetings over a period of 9 months and 7 optional sessions.<br>S: Individual and group sessions<br>P: A multidisciplinary team of therapists<br>M: - Sociotherapy based on transactional analysis and aimed at increasing social behaviour strategies<br>- Physiotherapy focused on graded activity including exercises and relaxation<br>- Psychotherapy with general information about fibromyalgia and pain mechanisms including methods of core qualities, rational emotive therapy, transactional analysis.<br>- Creative arts therapy<br>- Aftercare program to repeat key messages about coping<br>- Additional individual therapy sessions (optional)	**AE:**<br>D: Sessions from one hour, twice a week, during 12 weeks.<br>S: Group sessions<br>P: Physiotherapist<br>M: - Warm up with aerobic exercise and stretching<br>- Aerobic part<br>- Resistance training<br>- Cool down<br>- Home exercises<br><br>**CAU:**<br>D: One or two consultations<br>S: Individual sessions<br>P: Rheumatologist or specialized rheumatology nurse<br>M: - Individualized education about fibromyalgia<br>- Lifestyle advice<br>- Diversity of other treatments such as physiotherapy or social support from rheumatology nurse (optional)	- Perceived health (Visual Analogue Scale)	18 months	Moderate	- Perceived health increased in the MD group from 48.1 (1.7) at inflow to 57.3 (2.3) at 18 months after the program ($p < 0.05$). However, there was no significant difference between perceived health in the MD group and the CAU group. There was no significant increase in perceived health in the AE group.<br>- Hours of sick leave decreased significantly from 9.2 (1.0) to 1.2 (0.8) hours of sick leave 18 months after the program in the MD group ($p < 0.001$). Between the MD group and the CAU, the difference was not statistically significant. There was no significant decrease in hours of sick leave for the AE group.<br>- Contractual hours paid work did not change for the AE group or the MD group.
Vibe Fersum K. et al. 2013 (37) Norway (Europe)	Randomized controlled trial	**Classification-based cognitive functional therapy (CB-CFT):**<br>N: 51<br>A: 41.0 (10.3)<br>G: 24 males, 27 females<br>H: Non-specific chronic low back pain<br><br>**Manual therapy and exercise (MT-EX):**<br>N: 43<br>A: 42.9 (12.5)	**CB-CFT:**<br>D: Sessions from 30-60 minutes, weekly or ones every 2-3 weeks during 12 weeks<br>S: Individual sessions<br>P: Experienced physiotherapist<br>M: - Outlining the vicious cycle of pain based on findings from examination<br>- Movement exercises<br>- Targeted functional integration of activities in daily life<br>- Tailored physical activity program	**MT-EX:**<br>D: Multiple sessions of 30-60 minutes<br>S: Individual sessions<br>P: Specialized therapist in orthopaedic manual therapy<br>M: - Joint mobilization or manipulation technique for the spine or pelvis<br>- Exercises or a home exercise program including general exercise or motor control exercise (optional)	- Fear-avoidance beliefs (Fear-Avoidance Beliefs Questionnaire)	3, 12 months	Moderate	- Fear-avoidance beliefs for work decreased more for the CB-CFT group (from 14.1 to 8.3) than for the MT-EX group (from 19.1 to 17.4) after 3 months ($p < .001$). Fear-avoidance beliefs for work also decreased more for the CB-CFT group (from 14.1 to 7.7) than for the MT-EX group (from 19.1 to 16.6) after 12 months ($p < .001$). No information on significant within-group difference is provided.<br>- In the CB-CFT group number of people with more than 7 sick leave days changed from 23 at baseline to 10 at 12 months. No information on significant within-group difference is provided.<br>- The number of sick leave days after 12 months was lower in the CB-CFT group than in the MT-EX group after 12 months ($p < .01$), with 20.4% in the CB-CFT group with more than 7 sick leave days versus 42.5% in the MT-EX group with more than 7 sick leave days.

		G: 22 males, 21 females<br>H: Non-specific chronic low back pain						
Volker G. et al. 2017 (32) Netherlands (Europe)	Cohort study	N: 165<br>A: 44.1 (12.9)<br>G: 22 males, 143 females<br>H: Chronic musculoskeletal pain	**Standardized multidisciplinary team care intervention:**<br>D: 15 weeks<br>S: Individual and group sessions<br>P: Rehabilitation physician, occupational therapist, social worker, psychologist, physical therapist<br>M: - Cognitive behavioral therapy<br>- Education<br>- Individual and group exercises<br>- Relaxation<br>- Hydrotherapy	-	- Catastrophizing (Pain Catastrophizing Scale)	3, 12, 24 months	Moderate	- Catastrophizing decreased from a mean of 17.5 (9.6) at admission to 12.6 (9.1) at 24 months follow-up ($p < .05$).<br>- Number of patients that worked 0 or 1-24 hours decreased, whereas the number of patients working ≥ 25 hours a week increased ($p < .05$).
Wormgoor M. E. A. et al. 2020 (15) Norway (Europe)	Randomized controlled trial	**Brief psychotherapy (Brief-PsT)**<br>N: 141<br>A: 40.3 (10.9)<br>G: 45 males, 96 females<br>H: Common mental complaints<br><br>**Short-term psychotherapy (Short-PsT)**<br>N: 143<br>A: 42.9 (10.4)<br>G: 52 males, 91 females<br>H: Common mental complaints	**Brief psychotherapy (Brief-PsT)**<br>D: 6 sessions. First session 90 minutes and other sessions 50 minutes<br>S: Individual<br>P: Psychotherapists<br>M: - Psychotherapy sessions with focus on normalizing, accepting and coping with mental health complaints and their hindrance for work participation	**Short-term psychotherapy with more extended focus (Short-PsT)**<br>D: 20 sessions. First session 90 minutes and other sessions 50 minutes<br>S: Individual<br>P: Psychotherapists<br>M: - Psychotherapy sessions with focus on coping with mental health complaints and hindrance for work participation.<br>- Emphasis on an extensive anamnesis and possibility to establish a central theme based on previous or current challenging issues such as trauma or difficult childhood conditions<br>- Reducing symptoms and problematic behaviour and improvement of home situation, with deeper focus on cognitive maladaptive	- Self-efficacy (General self-efficacy scale)	3, 12, 24 months	Moderate	- Self-efficacy improved for participants in the Brief-PsT group from 2.6 at baseline to 3.1 at two-year follow-up and for participants in the Short-PST group from 2.6 at baseline to 3.0 at two-year follow-up ($p < .001$), but there was no difference over time between the two groups.<br>- Analyses showed statistically significant improved work participation (less days sick leave) in the Brief-PsT group ($p < 0.001$) and the Short-PsT group ($p < 0.001$). At 1-year follow-up, work participation was higher for the Brief-PsT group than the Short-PsT group ($p = .031$). At 2-year follow-up differences were not significant.

				coping strategies or dynamic repetitions				

*RTW: Return to work, *CAU: Care as usual
