# Supplementary material for: Interventions on cognitions and perceptions that influence work participation of employees with chronic health problems: a scoping review
Source: BMC Public Health. 2020 Oct 27;20:1610. doi: 10.1186/s12889-020-09621-5 (PMC7590449; doi:10.1186/s12889-020-09621-5)
Supplement: Supplementary file 3 — Additional file 3: Table 1. Risk of bias of randomized controlled trials. Table 2. Risk of bias of cohort studies. Table 3. Risk of bias of non-randomized experimental studies and studies with a single group pre-test post-test design. [file 12889_2020_9621_MOESM3_ESM.pdf]

Additional file 3

Table 1. *Risk of bias of randomized controlled trials*

Study	Q1	Q2	Q3	Q4	Q5	Q6	Q7	Q8	Q9	Q10	Q11	Q12	Q13	Quality score /total	Risk of bias
Aasdahl L. et al. 2019 (38)	1	0	1	0	0	0	1	1	0	1	1	0	1	7/13	Moderate
Arends I. et al. 2014 (43)	1	1	0	1	0	1	1	1	1	1	1	0	1	10/13	Moderate
Fauser D. et al. 2019 (24)	1	1	1	0	0	0	1	0	1	1	1	1	1	9/13	Moderate
Granviken F. et al. 2015 (39)	1	1	0	0	0	1	1	1	1	1	1	1	1	10/13	Moderate
Hampel P. et al. 2019 (16)	1	1	1	0	0	0	1	1	0	1	0	1	1	8/13	Moderate
Harris A. et al. 2017 (40)	1	1	0	0	0	0	1	0	1	1	1	1	1	8/13	Moderate
Hees H. L. et al. 2013 (12)	1	1	1	0	0	1	1	1	1	1	1	1	1	11/13	Low
Hutting N. et al. 2015 (13)	1	1	1	0	0	0	0	0	1	1	0	1	1	7/13	Moderate
Marchand G. H. et al. 2015 (41)	1	1	1	0	0	1	0	1	1	1	1	1	1	10/13	Moderate
Muschalla B. et al. 2016 (14)	0	0	1	0	0	0	1	1	0	1	1	1	1	7/13	Moderate
Pedersen P. et al. 2015 (23)	1	1	0	0	0	1	0	1	1	1	1	1	1	9/13	Moderate
Rolving N. et al. 2015 (29)	1	1	0	0	0	0	1	0	1	1	1	1	1	8/13	Moderate
Ronzi Y. et al. 2017 (42)	1	1	0	0	0	0	1	0	1	1	1	1	1	8/13	Moderate
Van Eijk-Hustings Y. et al. 2013 (25)	1	1	0	1	0	0	1	1	1	1	1	1	1	10/13	Moderate
Vibe Fersum K. et al. 2013 (37)	1	1	0	0	0	1	1	1	1	1	1	0	1	9/13	Moderate
Wormgoor M. E. A. et al. 2020 (15)	1	1	1	0	0	0	1	1	1	1	1	1	1	10/13	Moderate

Items Joanna Briggs Institute critical appraisal checklist for randomized controlled trials: Q1: Randomization, Q2: Allocation concealment, Q3: Similarity groups at baseline, Q4: Blinding participants, Q5: Blinding treatment providers, Q6: Blinding outcome assessors, Q7: Treatment groups treated identically, Q8: Completeness follow-up, Q9: Intention to treat analysis, Q10: Identical outcome measures, Q11: Reliability outcome measures, Q12: Appropriate statistical analysis, Q13: Appropriate trial design

1: Description in study meets criterion; 0: Description in study does not meet criterion

Table 2. *Risk of bias of cohort studies*

Study	Q1	Q2	Q3	Q4	Q5	Q6	Q7	Q8	Q9	Q10	Q11	Quality score /total	Risk of bias
Adams H. et al. 2017 (33)	1	1	1	1	1	1	1	0	1	X	1	10/11	Low
Asih S. et al. 2015 (44)	1	1	1	0	0	0	1	1	0	0	1	6/11	Moderate
Chu M. C. et al. 2015 (17)	1	1	1	1	1	0	1	1	0	0	1	8/11	Moderate
Gagnon C. M. et al. 2013 (31)	1	1	1	0	0	0	1	0	0	0	1	5/11	High
Haiduk P. et al. 2017 (27)	1	1	1	0	0	0	1	1	0	1	1	7/11	Moderate
Pietilä-Holmner E. et al. 2020 (26)	1	1	1	1	1	0	1	1	0	0	1	8/11	Moderate
Salzwedel A. et al. 2020 (19)	1	1	1	1	1	0	0	1	1	1	1	9/11	Low
Sullivan M. et al. 2017 (34)	1	1	1	1	1	1	1	0	0	1	1	9/11	Low
Volker G. et al. 2017 (32)	1	1	1	0	0	0	1	1	0	1	1	7/11	Moderate

Items Joanna Briggs Institute critical appraisal checklist for cohort studies: Q1: Similarity groups, Q2: Similarity exposure measurement, Q3: Validity and reliability exposure measurement, Q4: Identification confounders, Q5: Dealing with confounders, Q6: Participants free of outcome at start, Q7: Validity and reliability outcome measures, Q8: Sufficiency follow-up time, Q9: Completeness follow-up, Q10: Strategies for incomplete follow-up, Q11: Appropriate statistical analysis

X: Item not applicable; 1: Description in study meets criterion; 0: Description in study does not meet criterion

Table 3. *Risk of bias of non-randomized experimental studies and studies with a single group pre-test post-test design*

Study	Q1	Q2	Q3	Q4	Q5	Q6	Q7	Q8	Q9	Quality score /total	Risk of bias
Aasdahl L. et al. 2018 (28)	1	1	1	0	1	0	1	1	1	7/9	Moderate
Jensen A. G. C. 2013 (20)	1	1	0	1	1	1	1	1	1	8/9	Low
Leensen M. C. J. et al. 2017 (18)	1	1	1	0	1	0	1	1	1	7/9	Moderate
Scott W. et al. 2014 (30)	1	1	1	0	0	1	1	1	1	7/9	Moderate

Items Joanna Briggs Institute critical appraisal checklist for quasi-experimental studies: Q1: Clear cause and effect, Q2: Similarity groups, Q3: Similarity treatment, Q4: Presence of control group, Q5: Multiple measurements of outcome pre and post, Q6: Completeness follow-up, Q7: Similarity outcome measurement, Q8: Reliability outcome measures, Q9: Appropriate statistical analysis

1: Description in study meets criterion; 0: Description in study does not meet criterion
